# The anatomy and development of the nervous system in Magelonidae (Annelida) – insights into the evolution of the annelid brain

**DOI:** 10.1186/s12862-019-1498-9

**Published:** 2019-08-28

**Authors:** Patrick Beckers, Conrad Helm, Thomas Bartolomaeus

**Affiliations:** 1 Institute of Evolutionary Biology, University of Bonn, 53121 Bonn, Germany; 2 Johann-Friedrich-Blumenbach Institute for Zoology & Anthropology, Animal Evolution and Biodiversity, University of Göttingen, 37073 Göttingen, Germany

**Keywords:** Invertebrate, Evolution, Brain, Nervous system development, Larva

## Abstract

**Background:**

The annelid anterior central nervous system is often described to consist of a dorsal prostomial brain, consisting of several commissures and connected to the ventral ganglionic nerve cord via circumesophageal connectives. In the light of current molecular phylogenies, our assumptions on the primary design of the nervous system in Annelida has to be reconsidered. For that purpose we provide a detailed investigation of the adult nervous system of Magelonidae – a putatively basally branching annelid family - and studied early stages of the development of the latter.

**Results:**

Our comparative investigation using an integrative morphological approach shows that the nervous system of Magelonidae is located inside the epidermis. The brain is composed of an anterior compact neuropil and posteriorly encircles the prostomial coelomic cavities. From the brain two lateral medullary cords branch off which fuse caudally. Prominent brain structures such as nuchal organs, ganglia or mushroom bodies are absent and the entire nervous system is medullary. Our investigations also contradict previous investigations and present an updated view on established assumptions and descriptions.

**Conclusion:**

The comprehensive dataset presented herein enables a detailed investigation of the magelonid anterior central nervous system for the first time. The data reveal that early in annelid evolution complexity of brains and anterior sensory structures rises. Polymorphic neurons in clusters and distinct brain parts, as well as lateral organs - all of which are not present in outgroup taxa and in the putative magelonid sister group Oweniidae - already evolved in Magelonidae. Commissures inside the brain, ganglia and nuchal organs, however, most likely evolved in the stem lineage of Amphinomidae + Sipuncula and Pleistoannelida (Errantia+ Sedentaria). The investigation demonstrates the necessity to continuously question established descriptions and interpretations of earlier publications and the need for transparent datasets. Our results also hint towards a stronger inclusion of larval morphology and developmental investigations in order to understand adult morphological features, not only in Annelida.

**Electronic supplementary material:**

The online version of this article (10.1186/s12862-019-1498-9) contains supplementary material, which is available to authorized users.

## Background

In textbooks the central nervous system (*cns*) of Annelida is described as to consist of a dorsal brain with several commissures, in most taxa located in the prostomium, and nerves that encircle the mouth and connect the brain to the ventral ganglionic nervous system [1, 2]. This statement, however, is challenged by the results of recent phylogenomic investigations to unravel the evolution of Annelida [3–8]. Hence, these studies lead to two important conclusions: 1: Most of our assumptions on the structure and primary organization of Annelida so far results from comparative analyses of different representatives of derived annelid ingroups, i.e. subgroups of the Pleistoannelida. 2: Those taxa which turned out to be basally branching are those which were neglected in most classical studies. The latter especially applies to the presumable sister groups Oweniidae and Magelonidae, which putatively form a monophylum that is the sister group to all remaining annelids. Additionally, recent neuroanatomical studies of Oweniidae show that the anatomy of the nervous system of these worms differs tremendously from the textbook annelid’s nervous system [9]. Thus, the brain of Oweniidae is ring shaped, encircles the mouth and is confluent with the ventral nervous system. Concentrations of neurites (tracts) and neurons (ganglia) are not present, the nervous system is medullary [9–11]. These differences either result from transformations in the stem lineage of Oweniidae or show ancestral annelid conditions or a mixture of both. Detailed studies into the nervous system of their sister group Magelonidae might help solving this issue.

Magelonidae is a taxon of sessile annelids with a worldwide distribution. The so-called shovel-head worms inhabit sandy or muddy bottoms and possess ventro-laterally located palps which are used for the transport of food particles (detritus) into the mouth opening and may have a sensory function [12, 13]. Different tracts or commissures that are absent in the brain neuropil of Oweniidae, are described in Magelonidae as well as in pleistoannelid taxa [1, 2, 9]. Tracts or commissures are regions inside the neuropil of the brain, where neurites are differentially arranged compared to the neurites of the surrounding neuropil and thus can be discriminated. These tracts or commissures may connect certain regions of the brain like the sensory cells of the eyes; in that case the tract is called optical commissure or optical tract [14]. Orrhage [15] mentions four of such tracts for *Magelona papillicornis*, two of them being located in the dorsal and two in the ventral part of the brain. Orrhage used the course of these tracts to infer relationships between the different annelid taxa and to homologize the different head appendages, since these appendages are innervated by nerves connected to certain tracts (2 for review).

Also challenged by the “new” annelid phylogeny is the presence or absence of certain sensory structures or organs present in the last common ancestor of annelids. Among the prostomial ones, eyes and nuchal organs are the most prominent sensory structures [1, 16]. Nuchal organs are absent in Oweniidae and Magelonidae [9, 15, 17]. While larval eyes are described for *Owenia fusiformis* [18] and *Magelona mirabilis* [17], pigmented eye spots are only present in certain adult oweniids [9, 11] and are not present in adult Magelonidae. Despite these organs another sensory structure may be present in setigers of the trunk of Annelida. These ciliated grooves or papillae, located between neuro- and notopodium, are called lateral organs and are described for a variety of pleistoannelid polychaetes and the basally-branching Magelonidae, but are not present in Oweniidae [9, 16, 19, 20].

With the aim to add missing data to the information Orrhage [15] and McIntosh [21] provided for *Magelona papillicornis*, we studied the nervous systems of adult *Magelona mirabilis* and *Magelona alleni* using different morphological approaches, including serial sectioning, immunohistochemistry, μCT and transmission electron microscopy (TEM). To provide some insight into the development of the nervous system, we furthermore analyzed different early developmental stages of *Magelona mirabilis* using immunohistochemistry and subsequent confocal laser scanning microscopy (cLSM). During these studies, it turned out, that we were not able to follow all of the descriptions Orrhage provided for *Magelona papillicornis* [15]. Therefore, we applied different dyes (AZAN, Masson-Goldner, Palmgren’s silverstaining) to exclude that staining has an influence to the description of the brain anatomy. Additionally, the original sections Orrhage used for his 1966 work on *Magelona papillicornis* [15] were re-investigated and partly digitalized – and finally we were able to integrate Orrhage’s observations into a complete picture of the magelonid brain.

## Results

The information content of the Azan and Masson-Goldner-Trichrome stained sections (Additional file 1) is the same. For easier readability, we therefore describe the nervous system based on the Azan- stained section only. Nevertheless, both datasets were analysed to come to the conclusions made herein.

### The adult central nervous system

**Fig. 1:**
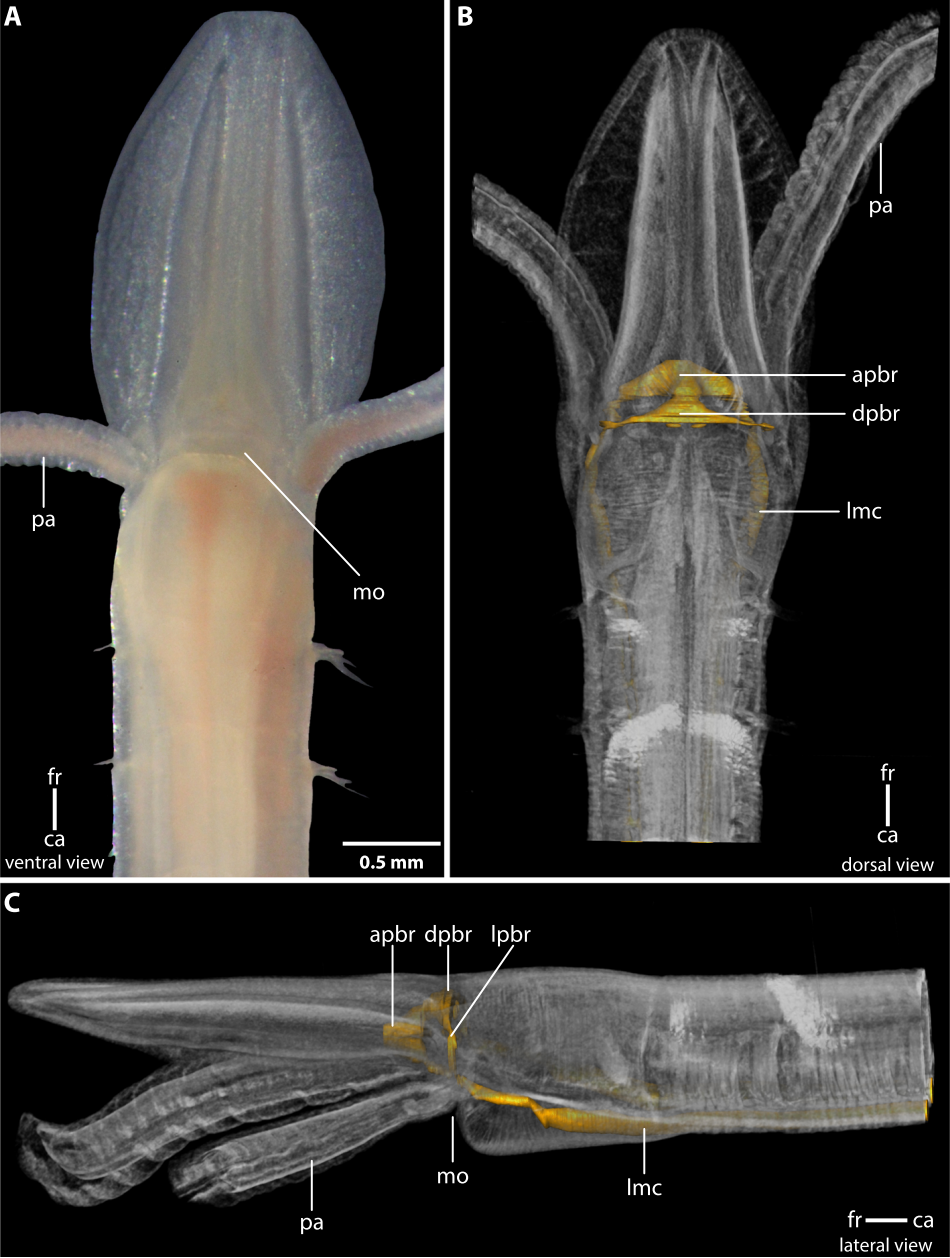
**a**: Living specimen of *Magelona mirabilis*. *ca*: caudal; *fr*: frontal; *mo*: mouth; *pa*: palp. **b**,**c**: Volume rendering of *Magelona mirabilis* and 3D-reconstruction of the central nervous system (*yellow*). *apbr*: anterior part of brain. *dpbr*: dorsal part of brain; *lmc*: lateral medullary cord; *lpbr*: lateral part of brain; *mo*: mouth; *pa*: palp

**Fig. 2:**
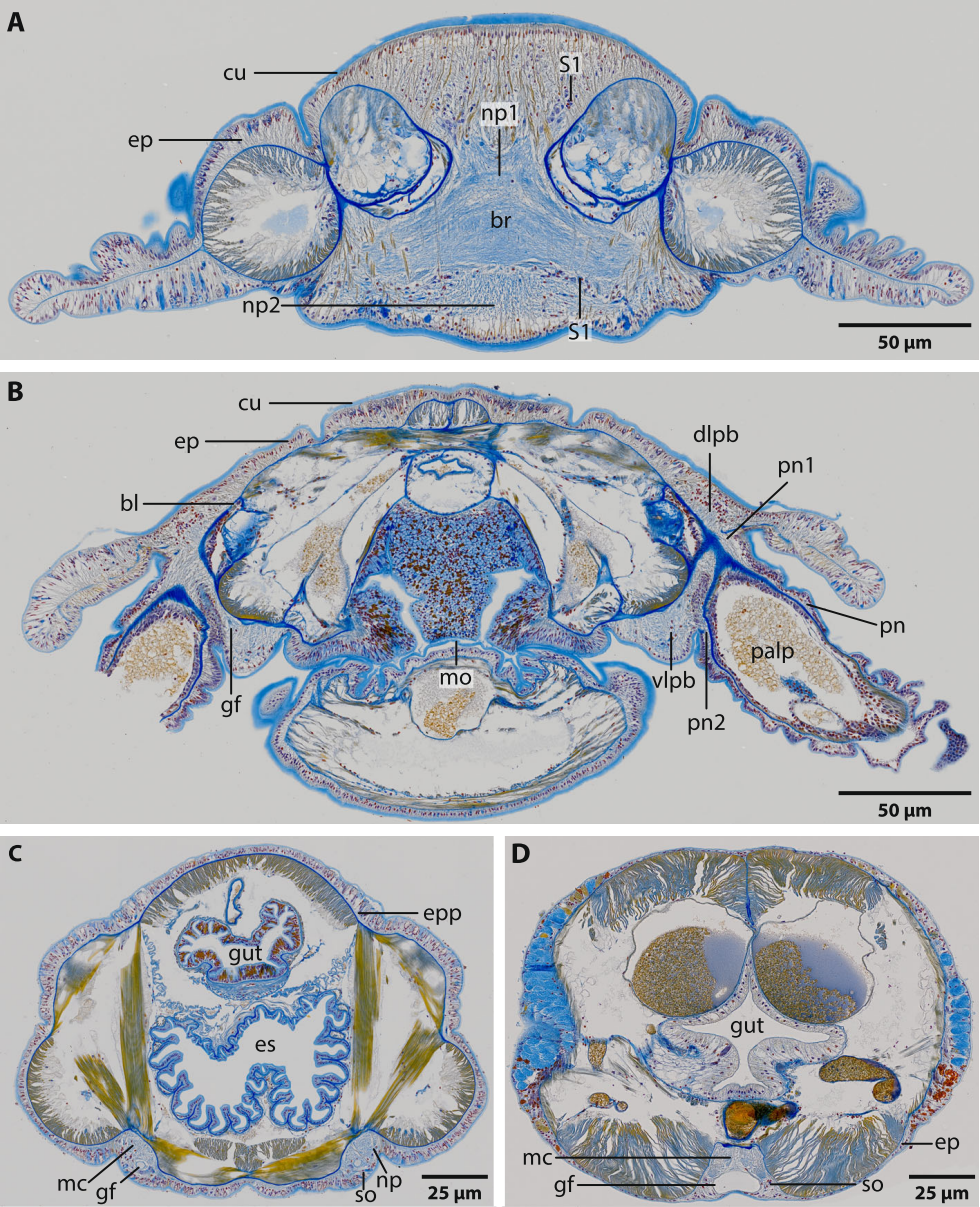
*Magelona mirabilis,* histological cross sections (5 μm), Azan staining, frontal (**a**) to caudal (**d**). **a**: The brain (*br*) of *M. mirabilis* is located inside the epidermis (*ep*). Frontally it is composed of two different types of neuropil (*np1*, *np2*). Neurites in *np1* are arranged in parallel to the body axis, while neurites in *np2* are arranged longitudinal to the body axis. Somata of the first type (*S1*) are located ventro- and dorso- laterally of the neuropil. *cu*: cuticle. **b**: Posteriorly the brain is composed of a dorsal and a ventral lobe. Both, the dorso- lateral (*dlpb*) and the ventro- lateral parts of the brain (*vlpb*) give rise to two nerves (*pn1*, *pn2*) which connect the palp nerve (*pn*) to the brain. *bl*: basal lamina; *gf*: giant fibre; *mo*: mouth opening. **c**: The brain gives rise to two laterally located medullary cords (*mc*). Somata (*so*) are located ventrally to the neuropil (*np*). An epidermal plexus (*epp*) is present and surrounds the whole animal. Both giant fibres (*gf*) can be traced to the ventro-lateral part of the brain. *es*: esophagus. **d**: Both medullary cords fuse ventrally to a single cord (*mc*) which is located in an epidermal invagination. Both giant fibres also fuse to a prominent single fibre (*gf*). Somata (*so*) are located ventro- laterally to the neuropil

**Fig. 3:**
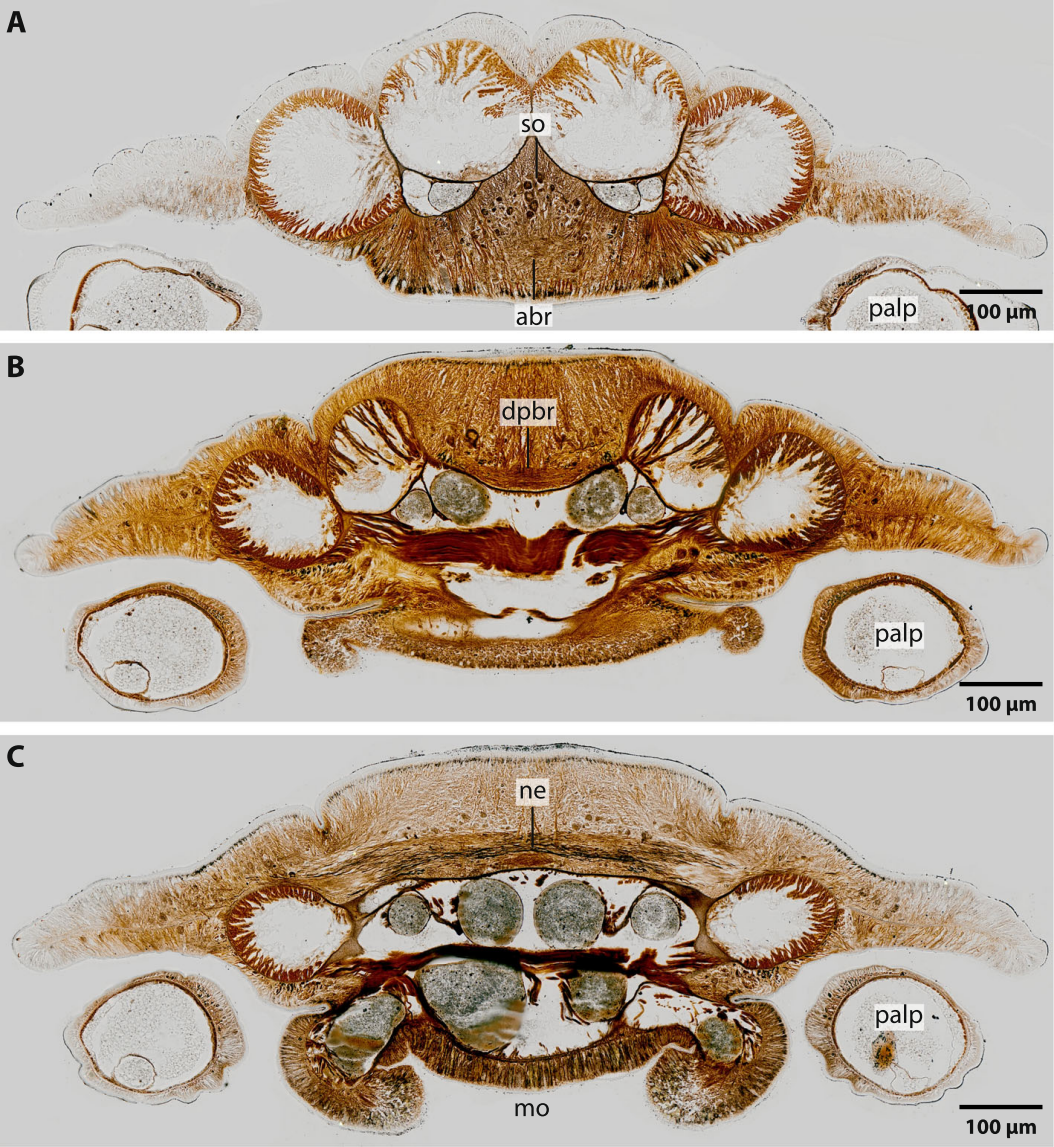
*Magelona mirabilis*, histological cross sections (5 μm), Palmgrens silver impregnation, frontal (A) to caudal (C). **a**: The anterior part of the brain (*abr*) is composed of a compact trapezoid neuropil. *so*: somata. **b**: Neurites in the dorsal part of the brain (*dpbr*) is composed of neurites which are arranged in parallel to the body axis. **c**: Some neurites are stronger impregnated by silver ions than the surrounding neurites. *mo*: mouth

**Fig. 4:**
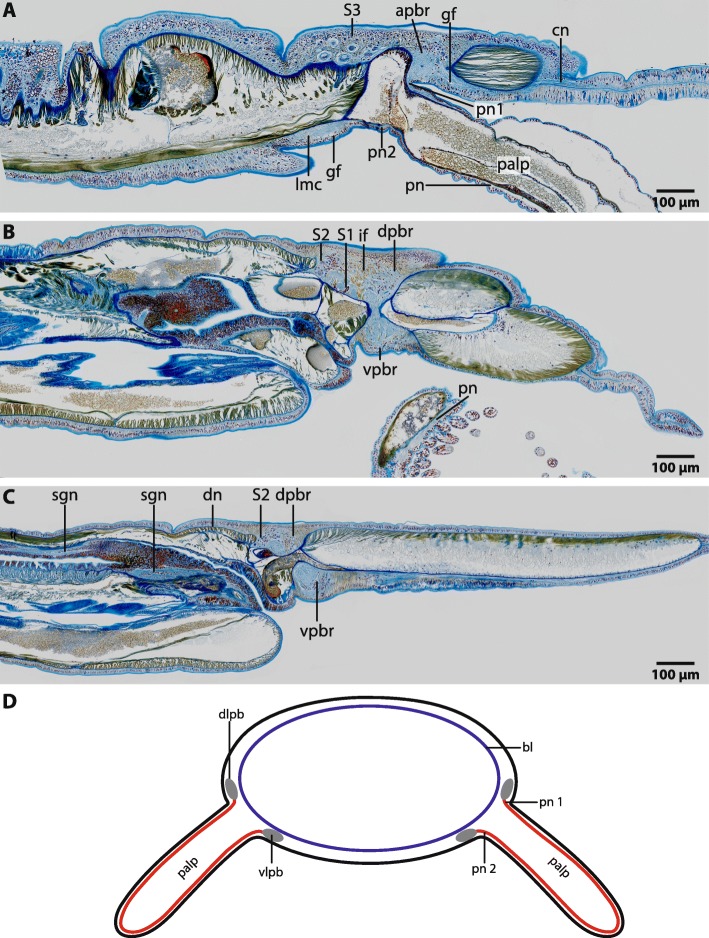
*Magelona mirabilis,* histological sagittal sections (5 μm), Azan staining **a**: The anterior part of the brain (*apbr*) is compact and gives rise to frontally located cephalic nerves (*cn*). A giant fibre (*gf*) originates in the ventral part of the brain and extends along the whole lengths of the lateral medullary cords (*lmc*). The palp nerve is connected to dorso- lateral and ventro- lateral part of the brain by two nerves (*pn1*, *pn2*). Neurons with very prominent somata (*S3*) are located posterior to the brain and are associated with the palp nerve. **b**: The dorsal part of the brain (*dpbr*) terminates in a layer of type two neurons (*S2*). A cluster of type one neurons (*S1*) is located between the neurites of the dorsal part of the brain. The palp is innervated by a basiepidermal palp nerve (*pn*). *vpbr*: ventral part of brain. Radial glia cells with prominent intermediate filaments (*if*) cross the neuropil. **c**: The dorsal part of the brain (*dpbr*) terminates in a layer of type two neurons (*S2*). A very delicate dorsal nerve originates in the neuropil of the posterior dorsal part of the brain (*dpbr*). The digestive system is innervated by stomatogastric nerves (*sgn*). *vpbr*: ventral part of brain. **d**: Palps are innervated by two nerves (*pn1*, *pn2*), of which one originates in the dorso- lateral (*dlpb*) and one in the ventro- lateral parts (*vlpb*) of the brain. *bl*: basal lamina

**Fig. 5:**
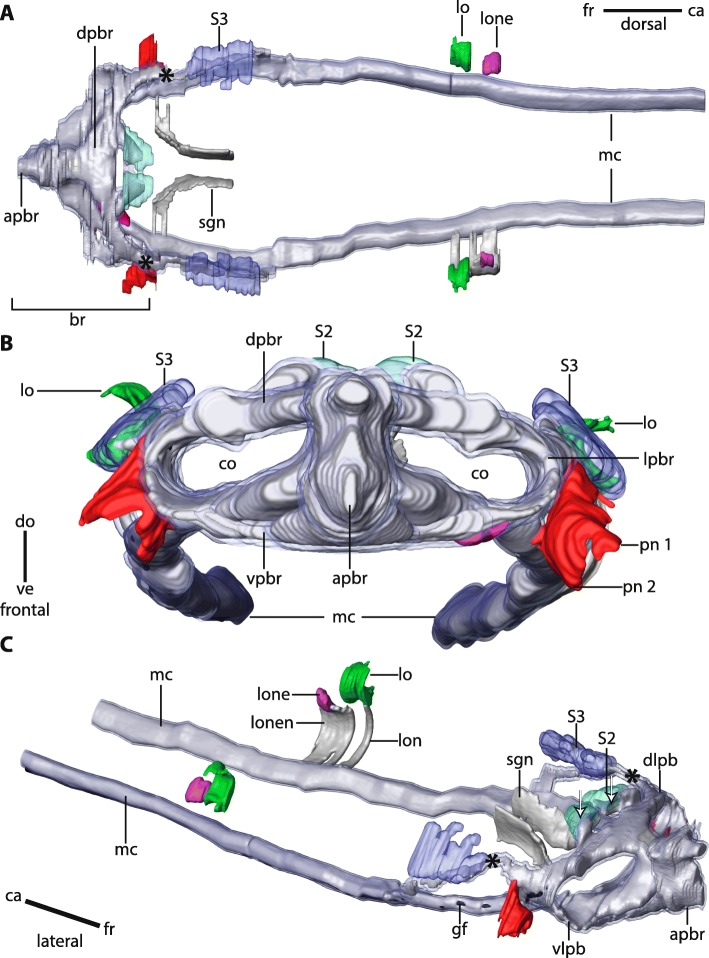
*Magelona mirabilis,* snapshots of 3D-reconstruction (485 sections, **a**: dorsal, **b**: frontal, **c**: lateral view). *black*: giant fibre; *bright blue*: neuronal somata type 1; *cyan*: neuronal somata type 2; *dark blue*: neuronal somata type 3; *green*: lateral organ; *grey*: neuropil; *purple*: neurons of the lateral organ; *red*: palp nerves. **a**: The anterior part of the brain (*apbr*) is compact. It is medially connected to the dorsal part of the brain (*dpbr*). The ventral part of the brain (*vpbr*) is confluent with the lateral medullary cords (*mc*). Caudally a lateral organ (*lo*) and enlarged neurons (*lone*) associated with the lateral organ are present. A cluster of neurons with very prominent cytoplasm (S3) is located dorsally to a nerve cord (*asterisk*) which originates in the vicinity of the palp nerve and merges with the lateral medullary cords (*mc*). *br*: brain. **b**: The anterior part of brain (*apbr*) is composed of a compact trapezoid neuropil in cross section. The ventral part of the brain (*vpbr*) is connected to the dorsal part of the brain (*dpbr*) by lateral brain parts (lpbr) which encircle the coelomic cavities (*co*). The lateral medullary cords (*mc*) are confluent with the ventral part of the brain (*vpbr*). Palps are innervated by two nerves (*pn1*, *pn2*). Different types of somata (*S2*, *S3*) are present. The lateral organ (*lo*) is located posterior to the brain. *S2*: neuron cluster type 2; *S3*: neuron cluster type 3. **c**: The giant fibre (*gf*) can be traced to the dorsal part of the brain and extends caudally along the medullary cords (*mc*). The stomatogastric system is innervated by nerves (*sgn*) which originate in the ventro-lateral parts of the brain (*vlpb*). The lateral organ (*lo*) is connected to the medullary cords (*mc*) by a lateral organ nerve (*lon*). A cluster of neurons with enlarged somata (*lone*) which is also connected to the medullary cord by a nerve (*lonen*) is associated with the lateral organ (*lo*). The dorso-lateral part of the brain (*dlpb*) gives rise to a nerve cord (*asterisk*) which fuses with the lateral medullary cord (*mc*). A cluster of very prominent neurons (*S3*) is located at the posterior end of this nerve cord. The dorsal part of the brain terminates in a cone shaped neuropil (*arrows*). *ca*: caudal; *fr*: frontal

**Fig. 6:**
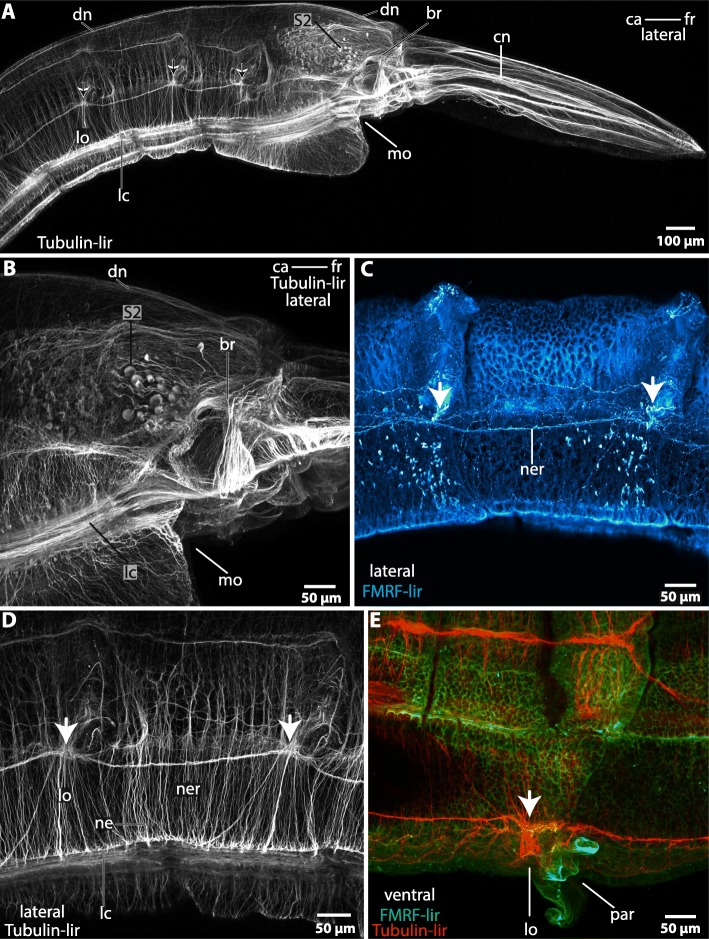
*Magelona mirabilis,* immunohistochemistry: **a**-**d** lateral view; **e**: ventral view. **a**: Tubulin-lir. A minor dorsal nerve (*dn*) arises in the posterior part of the brain (*br*). The tip of the head is innervated by cephalic nerves (*cn*) which originate in the brain (*br*). A lateral chain of neuropil concentrations (*arrows*) is connected to the brain. The concentrations correspond with the location of the lateral organ (*lo*). *ca*: caudal; *fr*: frontal; *mo*: mouth opening; *S2*: enlarged neurons. **b**: Tubulin-lir. In dorsal part of the brain (*br*) a cluster of enlarged neurons (*S2*) is present. The ventral part of the brain is confluent with the lateral medullary cords (*lc*). *ca*: caudal; *fr*: frontal; *mo*: mouth opening. **c**: FMRF-lir. Higher magnification of the lateral chain of neuropil concentrations in A. An accumulation of FMRF-positive neurites (*arrow*) is present at the heights of the lateral organ. The concentrations are interconnected by a nerve (*ner*). **d**: Tubulin-lir. Higher magnification of the lateral chain of neuropil knots in A. The ventro- lateral cords (*lc*) are connected to the chain of neuropil concentrations (*arrows*) by fine neurites (*ne*). **e**: Tubulin-lir, FMRF-lir, ventral view. The accumulations of neurites (*arrow*) are located close to the parapodia (*pa*) and correspond with the location of the lateral organ (*lo*). *ca*: caudal; *fo*: frontal

**Fig. 7:**
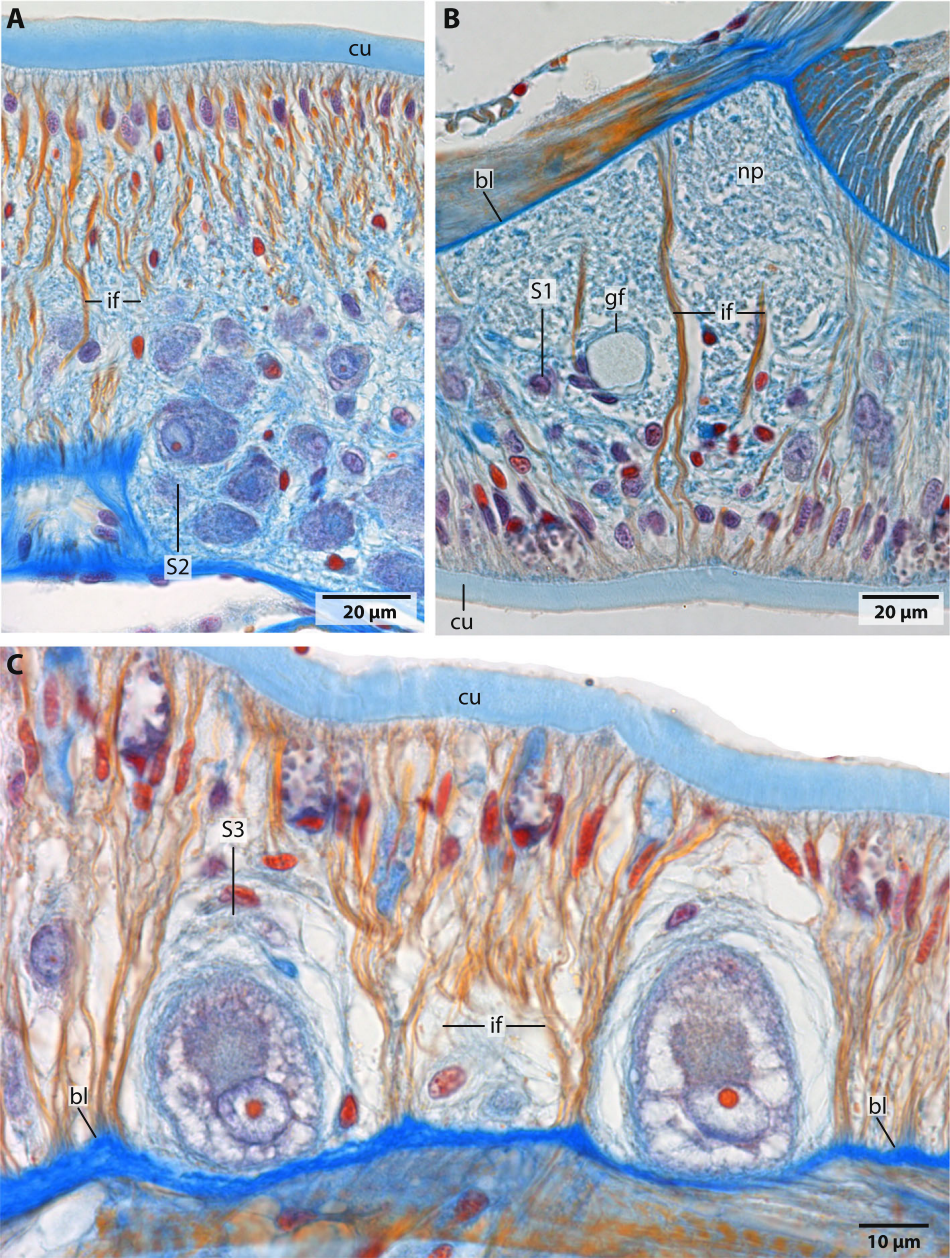
*Magelona mirabilis,* histological cross sections (5 μm), Azan staining. **a**: The posterior part of the dorsal neuropil of the brain neuropil terminates in a layer of enlarged neurons (*S2*). *cu*: cuticle; *if*: intermediate filaments. **b**: Intermediate filaments (*if*) are attached to the epidermal basal lamina and run through the neuropil (*np*) of the lateral medullary cords to be attached to the cuticle (*cu*). A giant fibre (*gf*) is present. S1: somata type 1. **c**: Glia cells with prominent intermediate filaments (*if*) are present in the epidermis. A cluster of neurons (*S3*) which possess very prominent cytoplasm around the nucleus is present in the dorso-lateral part of the brain and associated with the palp nerves. *bl*: basal lamina; *cu*: cuticle

The central nervous system (*cns*) of Magelonidae is located inside the epidermis (basiepidermal). The homogenous distribution of the neurons along the neuropil characterizes the *cns* as a medullary nervous system. The anterior part of the brain of Magelonidae consists of a central compact neuropil (Figs. 1b, c; 2a; 3a, 4a; 5; Additional file 3). This neuropil is located in the middle of the prostomium and fronto-laterally gives rise to two main neurite bundles. From these bundles numerous small neurites branch off and innervate the tip of the prostomium (Fig. 6a, b). Dorso- medially, the anterior brain neuropil is connected to the dorsal part of the brain (Figs. 1b, c; 2a; 3b, c; 4b; 5c; Additional file 3). The posterior dorsal part of the brain is slightly enlarged; it terminates in a medially located cone shaped neuropil which is coated by a cluster of enlarged neurons, here called S2 (Figs. 4b, c; 5; 6a, b; 7a; Additional file 3).

Ventrally of the compact anterior brain neuropil the brain expands and leads to the ventral part of the brain (Figs. 2b; 4b; 5; Additional file 3). The dorsal and ventral posterior parts of the brain are connected by laterally bulged crescent shaped lateral parts of the brain (Fig. 5b; Additional file 3). These parts of the brain encircle the paired protostomial coelomic cavities which extend to the tip of the prostomium (Fig. 5b, c; Additional file 3). The stomatogastric nervous system is innervated by paired nerves which originate in the ventro- lateral parts of the brain (Figs. 4c; 5a, c). Posterior to the brain, the central nervous system initially consists of two lateral medullary cords which extend caudally, rectangular to the brain (Figs. 2c; 4a; 5). The medullary cords are confluent with the brain; a subesophageal ganglion or distinct nerve roots are absent. The two medullary cords fuse at the heights of the midgut and run caudally as one single medullary cord. A giant fibre is located ventrally to the neuropil; somata are located ventro- laterally (Figs. 2d; 5c). Giant fibres can be traced to the dorso- lateral part of the brain (Figs. 2b; 4a). Anteriorly these fibres are small in diameter (approx. 10 μm) and follow the course of the two medullary cords and widen posteriorly (approx. 14 μm) (Figs. 2a, c; 5c; 7b). The two fibers fuse medio- ventrally prior of the fusion of the neuropils of the two medullary cords. From that point on, the giant fibre is very prominent and larger in diameter than the solitary fibres together (heights: 30 μm; width 80 μm) and is nearly as voluminous as the neuropil of the ventral cord (Fig. 2d).

### Head appendages

Adult *Magelona mirabilis* possess paired palps which are attached to the prostomium ventro- laterally (Figs. 1; 2b; 4a; 5). Palps are innervated by a basiepidermal nerve which surrounds the whole palp (Figs. 2b; 4b, d). The palp nerve is connected to the ventro-lateral and dorso-lateral parts of the brain by two nerves (Figs. 2b; 4a; 5). A nerve cord, which is associated with the type 3 neurons (S3) originates in the vicinity of the dorsal palp nerve and fuses caudally with the lateral medullary cords (Figs. 4a; 5; 7c). Conditions in early larval stages are described in a separate chapter below.

### The adult peripheral nervous system

Tubulin-lir immunoreactivity reveals a system of regularly arranged fine neurites which connect the main nerve cords and innervate the body integument and the epidermis (Fig. 6a, b, d). Additionally, there is a chain of neuropil concentrations which extends laterally along the whole lengths of the body. These neuropils are interconnected by small nerves which originate in the posterior part of the lateral region of the brain (Fig. 6c, d, e). The location of this chain corresponds with the location of the lateral organ (see section below). A small dorsal nerve, hardly detectable in Azan or silver staining, originates in the posterior part of the dorsal brain region and extends caudally (Fig. 6a). A basiepidermal nerve plexus is present in the trunk of the animal (Fig. 2c).

### Neurons

In Magelonidae investigated, three types of neurons can clearly be discriminated by size. The first type of neuron (S1) is compact; the cytoplasm surrounding the nucleus is not enlarged and cells resemble globuli cells (Figs. 2a; 3a, 4b; 5; 7b). These neurons are by far the most numerous found in investigated specimens. The cytoplasm of the second type of neuron (S2) is enlarged and dyes in purple (Figs. 4b; 5; 6a, b; 7a). These neurons appear in cluster in the dorso-caudal part of the brain (Figs. 4a; 5). The somata of the third type (S3) are more prominent than the somata of type 2 neurons and are only present on the dorso- lateral parts of the brain (Figs. 4a; 5; 7c). An unusual location of a type one neuron cluster is found in the center of the dorsal part of the brain (Fig. 4b). These neurons are not arranged on the outer surface of the neuropil but at the base of the basal lamina and extend into the neuropil (Fig. 4b).

### Glia

**Fig. 8:**
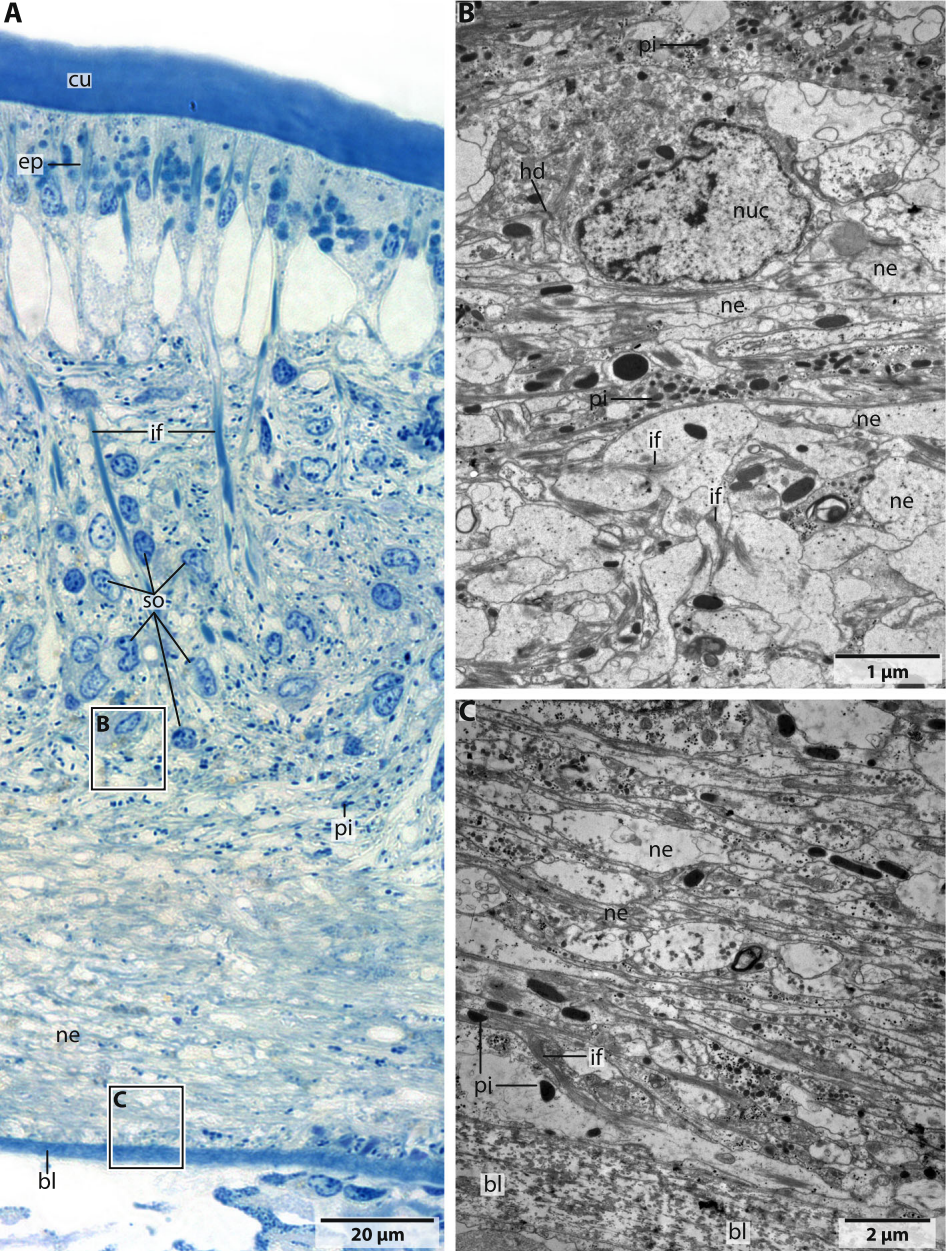
*Magelona mirabilis*, semi-thin section and ultrastructure. **a**: semithin section (1 μm) Toluidine blue; B,C: ultrathin sections (70 nm). A: The neurites (*ne*) of the nervous system are directly associated with the epidermal basal lamina (*bl*). Somata (*so*) of the neurons are located in the periphery of the neuropil. Radial glia cells which contain intermediate filaments (*if*) are attached to the basal lamina and run through the neuropil between the cells of the epidermis (*ep*). *cu*: cuticle. **b**: higher magnification of rectangle in A. The intermediate filaments (*if*) are attached by hemidesmosomes (*hd*) to the adjacent glia cells. Somata are located in the periphery of the neuropil. Neurites (*ne*) are differently orientated and pigment (*pi*) containing cells overlie the neuropil layer. **c**: higher magnification of rectangle in A. The neurites (*ne*) of the brain are located directly above the basal lamina (*bl*) of the epidermis. Pigment (*pi*) containing cells are less abundant than in the region towards the cuticle

The neurites of the brain neuropil are traversed by radial glia-like cells which contain prominent intermediate filaments (Figs. 4b; 7; 8). The respective cells are attached to the epidermal basal lamina and the cuticle by hemidesmosomes and cross the entire neuropil (Figs. 7; 8). In species investigated, glial cells do not form a layer or cortex around the neuropil and neurons. Additionally somata of the neurons are not enwrapped by glial cell processes (Fig. 8b).

### Ultrastructure

The neuropil consists of densely interwoven neurites which are directly associated with the epidermal basal lamina (Fig. 8). In *M. mirabilis* neurites of the brain are orientated in different directions. Neurites which innervate the tip of the prostomium are arranged in a longitudinal direction to the body axis of the animal, while the dorsally or ventrally located neuropils of the brain are composed of neurites which are arranged rectangular to the body axis (Figs. 2a; 3a, c). Neurites in the central nervous system are of different sizes (Fig. 8). In the dorsal and ventral regions of the brain the neurites are arranged in parallel to each other and are strongly impregnated by silver ions in the respective staining (Fig. 3c). In these regions the neurites appear to be more prominent than in the remaining *cns* (Fig. 8). Pigment containing cells are arranged as a layer above the neuropil of the brain (Fig. 8). Although we used different histological dyes as well as Tubulin-lir we did not find any signs of differentiated tracts or commissures in the brains of investigated specimen. We also found no tracts or commissures in the brain of *Magelona papillicornis* described by Orrhage (1966).

Higher brain centres like mushroom bodies or a glomerular neuropil were not found in the brains of investigated species.

**Fig. 9:**
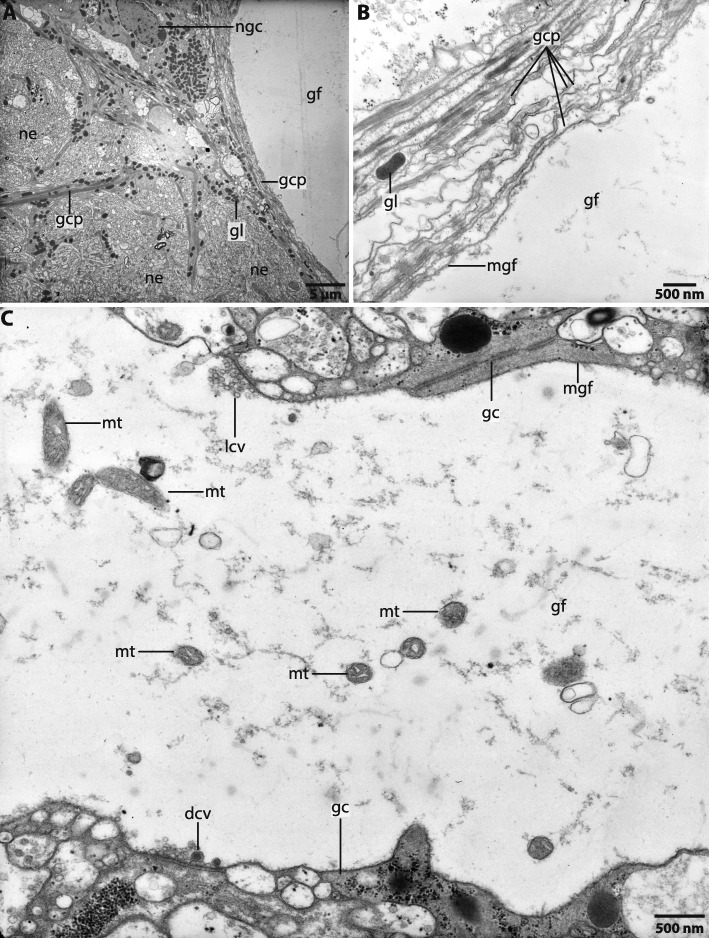
*Magelona mirabilis*, ultrastructure of the giant fibre. A-C 70 nm, **a**,**b** cross section, **c** sagittal section. **a**: the giant fibre (*gf*) is surrounded by a prominent layer of glia cell processes (*gcp*) which contain numerous gliosomes (*gl*). Nuclei (*ngc*) of the glia cells are located between the giant fibre and the neurites (*ne*) of the neuropil. **b**: The giant fibre (*gf*) is coated by a membrane (*mgf*) and separated from the neuropil by glia cell processes (*gcp*) which contain gliosomes (*gl*). **c**: The cytoplasm of the giant fibre (*gf*) contains numerous mitochondria (*mt*), lucent (*lcv*) and dense core vesicles (*dcv*). *gc*: glial cell; *mgf*: membrane of giant fibre

The giant fibre is coated by a membrane and surrounded by a prominent layer of glial cell processes which separate the giant fibre to the neurites of the central nervous system (Fig. 9a, b). Mitochondria and lucent and dense core vesicles indicating neurotransmitter are present inside the cytoplasm of the giant fibre (Fig. 9c). Membranes isolating the different cells of the giant fibres were not observed, thus the fibre is syncytial. We did not find any somata connected to the anterior part of the giant fibre. Nerve cell bodies of the giant fibres seem to be laterally scattered along the course of the fibre.

### Sensory structures

**Fig. 10:**
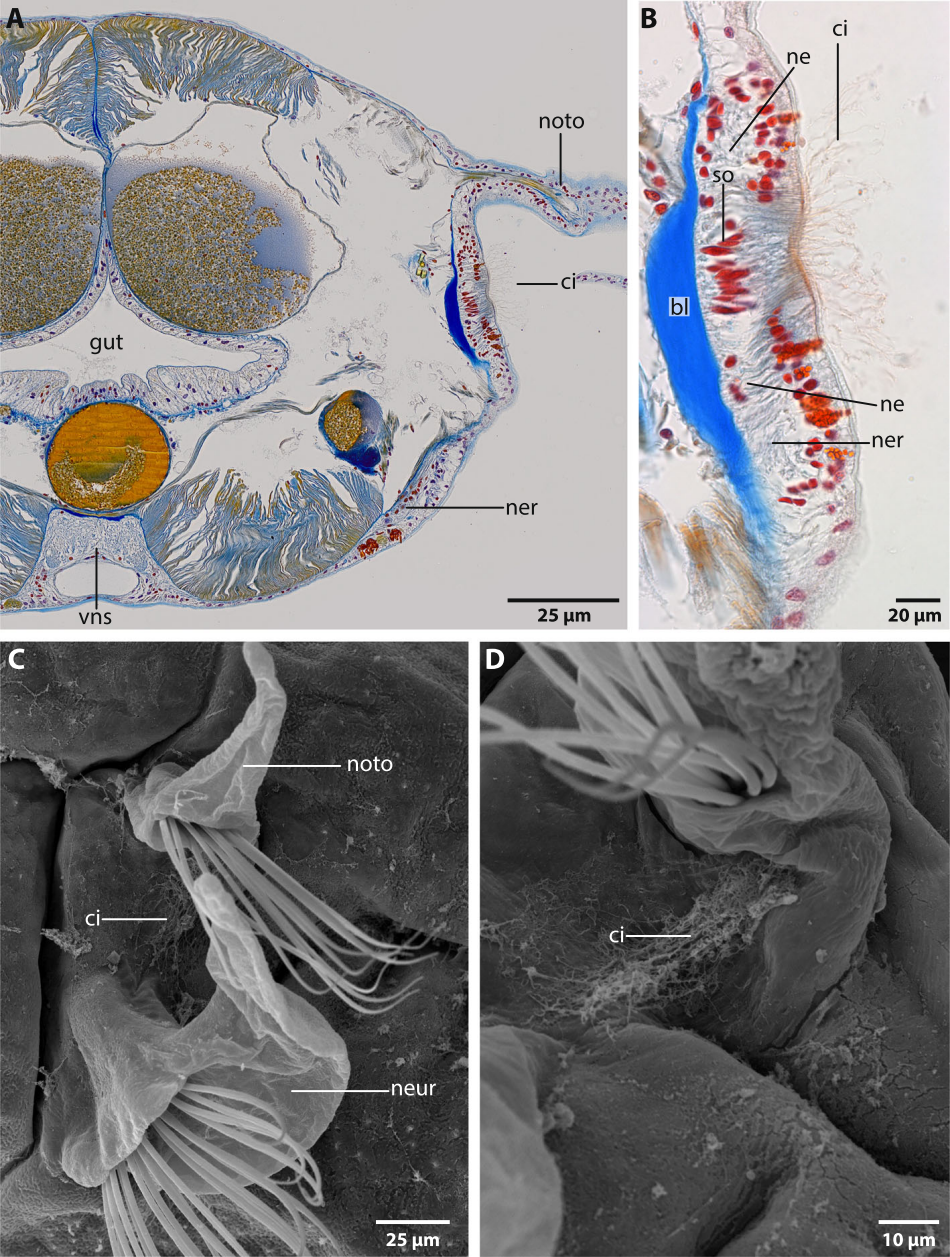
*Magelona mirabilis,* lateral organ. **a**,**b** histological cross sections (5 μm), Azan staining. **c**,**d**: SEM. **a**: The lateral organ is located between the neuro- and notopodium (*noto*). A nerve (*ner*) connects the sensory cells of the lateral organ to the ventral nervous system (*vns*). *ci*: cilia. **b**: Sensory cells which bear long cilia (*ci*) are connected to a nerve (*ner*) by neurites (*ne*). *bl*: basal lamina; *so*: somata. **c**: The lateral organ consists of a patch of elongated cilia between the neuro- (*neur*) and notopodium (*noto*). **d**: higher magnification of C: Cilia (*ci*) of the lateral organ are elongated

Between the neuro- and notopodium a densely ciliated pit is present in adult specimens (Fig. 10). Cilia in this region are elongated (Fig. 10b, c, d). Sensory cells are innervated by nerves which originate in the lateral medullary cords (Figs. 5a, c; 10b). Tubulin-lir reveals a chain of neuropil concentrations which are connected by a nerve which leads to the brain (Fig. 6). The location of this chain corresponds with the location of the lateral organ. A cluster of enlarged neurons (S2) is present in the vicinity of the lateral organ (Fig. 5).

Eyes and nuchal organs are absent in adult Magelonidae*.* Nevertheless, larval stages bear pigmented eyespots and anterior ciliary organs. A detailed investigation of these structures is not in focus of the present investigation and will be part of subsequent studies.

### The larval nervous system

For consistency and better understanding, we refer to the larval structures based on the vocabulary used in other publications. In case the respective structure is not observable in the adult *cns* with same characteristics the putative adult equivalents are given in brackets.

**Fig. 11:**
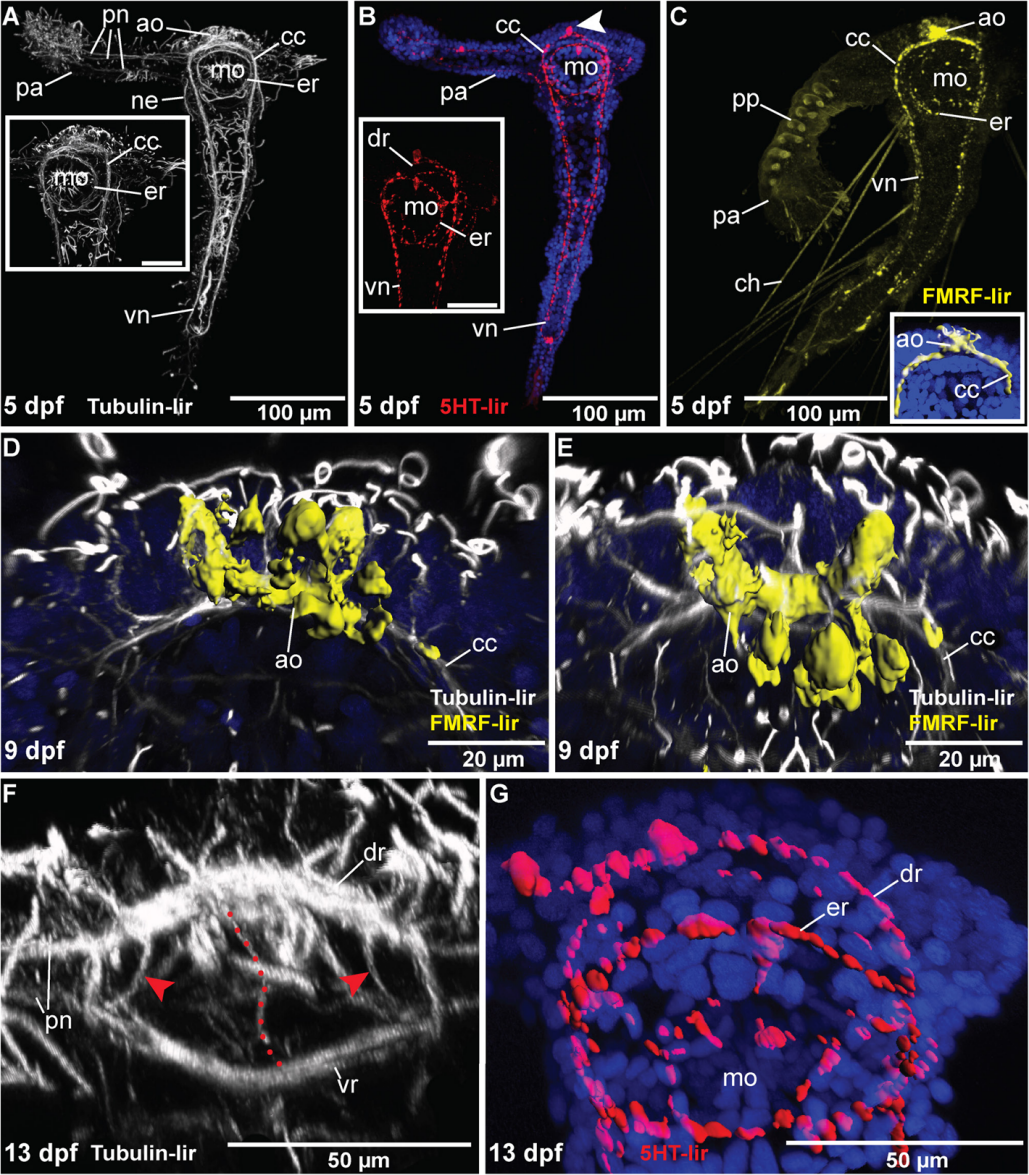
*Magelona mirabilis,* immunohistochemical patterns in larval stages*.* Confocal z-projections and volume renderings (**c** (inset), **d**, **e**, **g**). DNA staining with DAPI is shown in blue. Apical is up in all images except of (**e**) and (**f**) which show an apical view. Stages are given in days post fertilization (*dpf*). **a**: The nervous system of larvae around 5 dpf is characterized by the presence of well-developed neurites along the larval palp (*pa*) and along the ventral nerve cord (*vn*). The inset shows distinct circumesophageal connectives (*cc*) and a separate esophageal nerve ring (*er*). **b**: 5-HT-lir reveals the presence of a prominent apical soma (white arrowhead) and at least two neurite bundles forming the ventral nerve cord (*vn*). 5-HT-lir is present in the dorsal root of the circumesophageal connective (*dr*). **c**: FMRFamide-LIR is present in the apical organ (*ao*), the circumesophageal connectives (cc), in the esophageal nerve ring (*er*) and in the ventral nerve cord (*vn*). The inset shows a volume-rendering of the FMRF-lir in the apical region. **d**: The apical organ (ao) at 9 dpf is more developed than in the previous stages. A volume-rendering reveals the presence of at least five distinct somata showing FMRFamide-lir. **e**: The latter somata are located sagital and ventral of the circumesophageal connective (*cc*). **f**: An apical view of the anterior end at 13 dpf shows the dorsal (*dr*) and ventral root (*vr*) with outgoing palp nerves (*pn*) and distinct interconnections (red arrowheads, red dotted line). **g**: 5-HT-lir in the same stage reveals a prominent staining of the (*dr*) and the (*er*). The (*vr*) is only scarcely stained. *mo*, mouth opening; *ne*, nephridia; The scale bars only refer to the main images, not to the inserts

In early larvae of *Magelona mirabilis* (at 5 days post fertilization (*dpf*)) the nervous system is well developed and bears a ventral nerve cord arranged of two parallel neurite bundles, the esophageal nerve ring, prominent circumesophageal connectives (lateral parts of the brain), a well-defined apical organ and prominent nephridia in the anterior half of the body (Fig. 11a). Additionally, the larval palp is now obviously innervated by three neurite bundles originating from the circumesophageal connectives (lateral parts of the brain) and the dorsal and ventral root (dorsal and ventral parts of the brain) (Fig. 11a). 5-HT-lir is present along the ventral nerve cord, the circumesophageal connectives (lateral parts of the brain) and in the esophageal nerve ring (Fig. 11b). Notably, at least one prominent perikaryon showing 5-HT-lir is present in the apical region as part of the dorsal root of the circumesophageal connective (Fig. 11b). FMRF-lir at 5 dpf uncovers a prominent staining of the apical organ and the anterior part of the circumesophageal connective (Fig. 11c). Additionally, the ventral nerve cord shows distinct FMRF-lir (Fig. 11c).

At 9 dpf the earlier patterns observed for αTub-lir, 5-HT-lir and FMRF-lir in larvae at 5 dpf remain preserved. Apparently, the -lir of the apical organ changes significantly in this stage. Thus, FMRF-lir at 9 dpf reveals the presence of at least five distinct perikarya contributing to the apical complex and forming the apical organ (Fig. 11d, e).

At 13 dpf, the -lir is similar to the previous stage, but in contrast to earlier stages, a frontal view of the apical region reveals the presence of the dorsal and the ventral root of the circumesophageal connective and the lack of an apical nerve ring (Fig. 11f). Additionally, both roots (dorsal and ventral parts of the brain) are interconnected via lateral neurites and one median nerve (Fig. 11f). 5-HT-lir is present along the dorsal root of the circumesophageal connective, along the esophageal nerve ring and around the mouth opening (Fig. 11g). Observations in later stages were not possible due to the high mortality of the larvae probably caused by a change of lifestyle and food source.

In larvae (5 dpf – 13 dpf) the single palp is innervated by three main neurite bundles and connected to the dorsal and ventral root of the esophageal connective (Fig. 11a, f). Notably, the larval palp originates from the region of the equatorial ciliated band in older larval stages. Whether the adult and larval palps are homologous structures is still debated and will be part of the discussion.

### Orrhages reconstructions of the nervous system in adult *Magelona papillicornis*

**Fig. 12:**
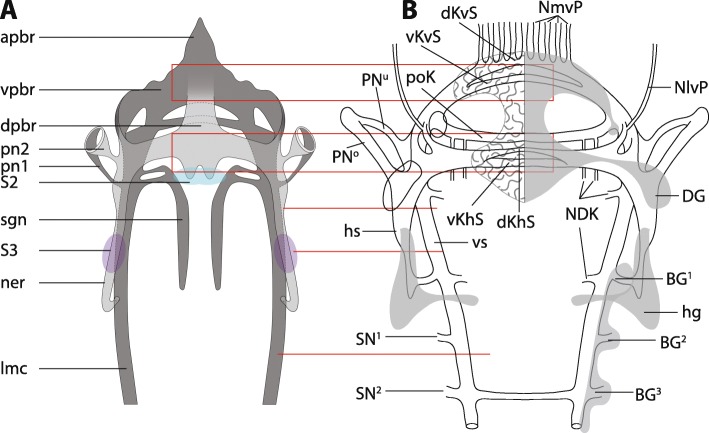
Comparison of Orrhage’s 1966 [15] schematic drawing of the nervous system of *Magelona papillicornis* (**b**) and our results of *Magelona mirabilis* (**a**). The red squares and lines indicate regions with disparities. A: schematic drawing after 3D-reconstruction. Somata of the first type were omitted. *Apbr*: anterior part of the brain; *dpbr*: dorsal part of the brain; *lmc*: lateral medullary cord; *ner*: nerve cord; *pn1*: palp nerve 1; *pn2*: palp nerve 2; *S2*: somata of the second type; *S3*: somata of the third type; *sgn*: stomatogastric nerves; *vpbr*: ventral part of the brain. **b**: Orrhages schematic drawing of the nervous system of *Magelona papillicornis*, redrawn from [15], for abbreviations see [15]

Although we carefully re- investigated sections of adult *Magelona papillicornis*, we were not able to discern the different tracts (*vKhS*, *dKhS*) Orrhage described in the brain, neither in his sections nor in ours (Fig. 12; Additional file 2). In his schematic drawing the prostomium is innervated by nerves which originate in the dorsal commissure of the brain. These nerves, however, originate in the anterior compact part of the brain (Fig. 12). He describes a dorsally located ganglion in the brain of *M. papillicornis* (*DG* in Orrhage 1966) (Fig. 12). This cluster of neurons is not present in investigated magelonids. The cluster of neurons Orrhage called *hg* (“plasma- rich ganglionic cells”; S3 in Figs. 5; 7c) (Fig. 12) is an accumulation of neurons with very prominent somata. He also found ganglia (BG1-BG3 in Orrhage 1966) (Fig. 12) in the lateral medullary cords of *Magelona papillicornis*. These ganglia are neither present in *Magelona mirabilis* nor in *Magelona alleni*, even though different methods were used to check for their presence. The description of the innervation patterns of the palps is in congruence with our observations that two nerves innervate the palps in adults (PN° and PN^u^ Fig. 12b) [15]. In his descriptions, the lateral medullary cords are connected to the brain by two nerves (hS and vS, frontal esophageal connective and posterior esophageal connective). He interpreted this as two roots of the circumesophageal connectives (*cc*). However, different roots of the *cc* do not exist in *Magelona mirabilis* or *M. alleni* (see discussion).

## Discussion

### Comparison with adult Oweniidae and outgroup taxa

The *cns* of Magelonids is located inside the epidermis (intraepidermal). The neuropil layer appears attached directly to the epidermal basal lamina. The ventral nervous system initially consists of lateral medullary cords which are located rectangular to the brain, but fuse in their further course to a single medullary cord. Ganglia or somata-free connectives are not present. The same is true for the putative sister taxon Oweniidae [8, 9], indicating that an intraepidermal *cns* with lateral medullary cords which fuse caudally might be the plesiomorphic condition for Annelida.

Contrary to the similarities found for the ventral nerve cord, the brain of magelonids is more complex in terms of neuron types. The anterior brain neuropil in magelonids is compact and the dorsal part of the brain is enlarged when compared to the brains of Oweniidae. In oweniids the brain is a simple ring surrounding the mouth (Beckers et al. 2019), while in magelonids the anterior brain is compact and posteriorly encircles the prostomial coelomic cavities. However, compared to the brain of errant polychaetes [1], the *cn*s of Magelonidae is rather simple. There are no ganglia present, neither in the anterior *cns* nor in the ventral nervous system. The whole *cns* is medullary. No tracts such as ventral or dorsal commissural tracts described by Orrhage for *M. papillicornis* [15] were found; neurites in the neuropil are more or less homogenous. Neither glomeruli in the brain as described for errant polychaetes [14, 22] or hoplonemerteans [23] nor mushroom bodies [1, 24] were found during our investigation. In Oweniidae only one type of neurons is known within the *cns* [9]. This neuron type is defined by the very little cytoplasm surrounding the nucleus and is also the most abundant type in Magelonids. However, in *Magelona mirabilis* and *M. alleni* a second and third type of neuron is present, which are located in the dorsal or dorso- lateral parts of the brain. The cytoplasm of these neurons is enlarged in comparison to the first type, whereas the second one is smaller than the third one. Furthermore, these neurons appear in clusters. Clustered polymorphic neurons are also found in Amphinomidae and Sipuncula as well as in pleistoannelid taxa [1]. However, polymorphic neurons are not described for Chaetopteriformia [1], which form another basally-branching clade within the annelid tree and therefore hamper the explanation of the evolution of these neuron types [8].

In basally branching taxa of the putatively outgroup taxa Nemertea and Mollusca the *cns* is also medullary and the brains of these taxa are more or less circular shaped. Polymorphic neurons and ganglia are not present [25, 26]. The same is true for Bryozoa, Brachiopoda and Phoronida [27–29]. Accordingly, the anatomy of the *cns* of spiralian relatives shows more similarities to the adult anatomy of Oweniidae and questions the sister-group relationship of oweniids and magelonids [8].

One might wonder why the nervous system of Magelonidae is more complex than the brain of the sister taxon Oweniidae, although adults of both taxa are sedentary and feed on detritus [30]. We can only speculate and suggest that the life style of the larva in magelonids might play a role in this respect. Thus, the larva of Magelonidae is predatory and actively feeds on bivalve larvae at least for 7 month in the plankton [31] and might need a more sophisticated nervous system for hunting. The larva of *Owenia fusiformis*, however, passively feeds on algae [32].

### Peripheral nervous system

A small dorsal nerve, as described for several other annelids [1], originates in the posterior dorsal part of the brain and extends caudally inside the epidermis. Since a dorsal nerve is not present in Oweniidae [9] a convergent evolution or a consecutive branching of oweniids and magelonids has to be assumed. Since there is no nuchal organ, the assumption that this nerve innervates the latter, seems not likely (see 1 for review).The epidermis is innervated by a dense network of mainly dorso- ventrally orientated neurites. These neurites connect the lateral medullary cords and the dorsal nerve like in other investigated annelids [1, 2]. An epidermal plexus as described for Oweniidae [9] is also present, indicating the plesiomorphic condition for Annelida.

### Sensory structures

In previous investigations, a lateral organ was mentioned to be present in adult specimens of *Magelona mirabilis* [19, 20], but only few details were known. It is composed of a densely ciliated spot between neuro- and notopodium. The spatial location and general anatomy resembles the lateral organs described for other polychaetes [19]. The evolutionary considerations behind that finding are difficult to evaluate with present knowledge, since Oweniidae as well as Chaetopteridae lack such an organ, but Apistobranchidae and Amphinomidae (both part of the radiation outside the Pleistoannelida) and at least some Pleistoannelida possess such an organ [19]. Further ultrastructural investigations are needed to clarify the evolutionary origin and value of the lateral organ in Annelida, because the dataset especially for basally-branching taxa is still quite scarce.

Nuchal organs are absent in adult and juveniles of Magelonidae thus far investigated (16; this study). Since these organs are also absent in outgroup taxa as well as in Oweniidae and Chaetopteriformia [9] this complex sensory organ evolved most likely in the stem lineage of Amphinomidae/Sipuncula and Pleistoannelida.

Adult stages of Magelonidae do not possess pigmented eyes, although they are present in larval stages. This situation is comparable to the situation in e.g. *Owenia fusiformis*, where the larva bears pigmented eyes of the cup- shaped type, while adults only possess rows of pigmented cells [9]. Why eye complexity or even the complete eyes are reduced during ontogenesis of these species remains unclear.

### Palps

Palps of adult Magelonidae are located ventro- laterally and palp nerves are connected to the dorso- and ventro- lateral parts of the brain. Notably, the palps of the basally branching oweniid taxon *Myriowenia* sp. are also innervated by two nerves each – in this case- originating in the dorsal and dorso- lateral part of the brain [9]. Nevertheless, we assume that the palps and palp nerves of both taxa are homologous and that spatial differences concerning the innervation points of the palp nerves in the brain might be caused by different head morphologies of both annelid taxa [9]. Two nerves also innervate the palps in *Magelona papillicornis*, Spionidae and Chaetopteridae [15], indicating that this pattern resembles the plesiomorphic condition in Annelida. Nevertheless, further investigations including more taxa spread all over the annelid tree are needed to investigate the evolution of palp innervation or general homology of palps throughout annelids.

### Glial cells

Glial cells contain intermediate filaments which classifies these cells as radial glia [33]. Furthermore, elongated cells traversing of the epithelium and basal endfeet in contact with the basal lamina, are characteristics of these cells. Radial glia seems to be especially important in species with an intraepidermal nervous system. These cells maintain the structure of the epidermis with an underlying nervous system. Although radial glial cells can be ascertained, a prominent glial layer surrounding the neuropil and somata as described for errant polychaetes [1] is not present. Since a prominent glial layer is suggested to protect the nervous system against mechanical distortions [33, 34] we suggest that the nervous system of magelonids is not that subjected to mechanical stress as the nervous system of e.g. errant species. Glial cell processes which wrap the neuronal somata are also not present due to our investigations.

### Giant fibre

Giant fibres are supposed to be involved in rapid signal conduction [35, 36] and are described for a variety of Pleistoannelida [1, 14] and for the putative sister taxon *Myriowenia* sp. (Oweniidae) [9]. The structure of this fibre in *Magelona mirabilis* resembles the anatomy of this fibre in *Myxicola infundibulum* (Sabellidae, Pleistoannelida) [37, 38], Siboglinidae [39, 40] and *Lumbricus terrstris* (Clitellata, Pleistoannelida) [36] indicating that a giant fibre was already present in the last common ancestor of Annelida. However, the giant fibre in Magelona is not composed of two intersecting large axons with anterior located somata as is the one in Myxicola [37]. Somata are most likely scattered along the course of the fibre. Whether giant fibers present in other invertebrate taxa are homologous remains unknown [41] but the absence of these fibres in nemerteans and platyhelminthes as well as in Brachiopoda and Bryozoans hint on a convergent evolution. Giant fibres in Octopus (Cephalopoda, Mollusca) [42], must have evolved convergently, since Polyplacophora and Solenogastres (Mollusca) lack such a fibre type.

### Comparative analysis of Orrhages reconstructions of the nervous system in adult *Magelona papillicornis* and this study

In his work on the nervous system of sedentary polychaetes, Orrhage described the anatomy of the brain in *Magelona papillicornis* based on histological sections [15] (Additional file 2). However, since *Magelona papillicornis* is originally described from Brazil, Fiege et al. [43] doubt the presence of this species in Europe and suggest that this species is not *Magelona papillicornis*, but most likely *M. mirabilis* [43]. In order to compare the results of Orrhage with our data, we re- investigated and digitalized the original sections he used for his schematic drawings of his 1966 paper [15]. Information content of his sections and staining are of the same quality like ours (Additional file 2). However, not all of his descriptions are in congruence with our observations. Although we carefully investigated the anatomy of the neuropil of the brain with different methods, we did not find any of the four commissures described by Orrhage (dKvS, vKvS; dKhS, vKhS in [15]) (Fig. 12). Instead, the neurites of the brain neuropil are homogenously arranged. Additionally, he did not recognize the medullary nature of the nervous system and found several ganglia. So, he wrongly described a cluster of neurons in the dorso-lateral part of the brain (DG in Fig. 12b) but did not mention the cluster of polymorphic neurons in the median dorsal part of the brain (S2 in Fig. 12a). The finding of the cluster of neurons with very prominent somata is validated (S3 in Fig. 12a; *hg* in Fig. 12b). Additionally, his observations on the innervation of the palp correspond to our results. Orrhage mentioned that the lateral medullary cords are connected to the brain by two nerves (hS and vS, frontal esophageal connective and posterior esophageal connective; Fig. 12). He interpreted this as two roots of the circumesophageal connectives (*cc*). However, this arrangement differs from the anatomy of roots of the *cc* in e.g. Eunicida. In these taxa the *cc* split close to the brain into two or more nerves (roots) which are connected to different commissures in the brain [2]. Since commissures in the brain of adult Magelonidae are absent and the connection of the second nerve cord leading to the *cc* is located more posterior and associated with the palp nerve, it is difficult to give any homology assumptions of this part of the brain to the different roots of the *cc* described for Errantia (Pleistoannelida). As shown in our investigations, different brain commissures forming roots inside the neuropil or a circumesophageal connective are only observable in larval stages of magelonids. Due to the medullary nature of the whole central nervous system in adult magelonids, terms like circumesophageal connectives have to be avoided.

### Comparative larval neuroanatomy

Based on our analyses the ontogenesis of the larval neural structures is comparable to conditions observed in other annelid larvae. Thus, a well-developed apical organ is present in magelonid larvae, possessing flask-shaped FMRFamidergic perikarya as well as serotonergic cells both early in development and throughout the entire larval ontogenesis. Comparable apical cell clusters showing both types of immunoreactivity can also be observed in most annelid larvae (Bleidorn et al. 2015), but serotonergic somata are absent in the putatively closely related early branching annelid *Owenia fusiformis* [18]. Notably, this lack of apical serotonergic somata is supposedly caused by the unusual larval shape and related re-arrangement of neural structures in larval Oweniidae.

Besides the developmental similarities to various annelid taxa, the larval magelonid nervous system is also well comparable to that of other spiralians. Even though the annelid sister group is not resolved yet, potentially closely related groups are represented by the Mollusca, Nemertea, Platyhelminthes, Phoronida and Brachiopoda [44, 45]. Prominent serotonergic and FMRFamidergic perikarya are also present in the larval apical organ of Polyplacophora [46, 47], Scaphopoda [48], Solenogastres [49], Brachiopoda [50] (shown only for serotonin), Phoronida [51] and Nemertea [25] and are also reported for Entoprocta and Platyhelminthes [51–54]. Absence of serotonergic somata has been only reported for Ectoprocta, so far [55] (and for Oweniidae). Thus, apical presence of 5-HT and FMRFamide-lir seems to be a plesiomorphic spiralian characteristic.

Furthermore, all the latter taxa, as well as the observed Magelonidae and most other annelid larvae, also develop at least two main nerve cords with numerous commissures and connection to the apical neuropil in early stages, and possess a distinct nerve ring surrounding the mouth opening [1]. Instead, a nerve ring underlying the prototroch is lacking in investigated magelonids.

The occurrence of different roots of larval brain commissures and the presence of two ventral nerve bundles early in the development hint towards an early onset of adult structures even in early larval stages of these annelids, in agreement with comparable structures that are known for adult nervous systems of other Annelida (Orrhage and Müller, 2005). There are two explanations for this situation: on the one hand we can argue that developmental investigations herein show a reduction of neuronal complexity from larval towards adult conditions. Although larval brain roots and well-developed circumesophageal connectives are present in early magelonid stages and well-known for the anterior adult neuroanatomy of different pleistoannelid taxa [1, 2], comparable structures are absent in adults of *M. mirabilis*. Just a dorsal and ventral part of the brain can be examined in this case. Similar conditions are obvious in oweniids – larval stages possess several brain commissures whereas adult specimens bear a simple ring-shaped brain without distinct roots [9, 18].

On the other hand, these findings can be interpreted in a different way: brain commissures can, per definition, only be present, if they are part of a compact brain neuropil. Any tracts that are found in different parts of the brain and that are not part of a uniform neuropil do thus not form a commissure. Since there is no compact mass present in tubulin- lir immunostainings in larval stages of *Magelona mirabilis*, we can also suggest that the dorsal and ventral part of the brain (including the roots) present in larval stages are retained in the adult stage and that the anterior compact neuropil develops later during ontogenesis. Additionally, the innervation of the palp from both brain parts (dorsal and ventral), like in the larvae, also hints towards the scenario that this larval brain may be part of the adult brain which surround the coelomic cavities. Such an ontogenetic transformation of the larval morphology with retention of the general assemblage concerning the respective brain areas in adult magelonids might be an explanation for the described conditions.

Nevertheless, one has to keep in mind that different brain commissures as described for the larval brain neuropil in several Pleistoannelida [2] are not present in all taxa, e.g. not in *Capitella telata* [56].

### Evolution of the nervous system in Annelida

The anatomy of the anterior nervous system of Magelonidae shows several characters comparable with the neuroanatomy of the closely related Oweniidae and possible annelid outgroup taxa as well as with the complex brains of pleistoannelid taxa. Thus, the intraepidermal position of the entire *cns* and the lack of ganglia presents the plesiomorphic annelid condition, which can also be investigated in annelid sister groups and in other basally-branching annelid taxa. On the other hand the enlargement of the dorsal part of the magelonid brain, the compact anterior neuropil and the presence of clusters of polymorphic neurons are characters only present in more derived annelid groups. Additionally a lateral organ is present, which is lacking in Oweniidae but present in several other derived Annelida. In contrast nuchal organs, complex cup-shaped eyes must have developed in the stem lineage of Sipunculida+ Amphinomidae and Pleistoannelida (Errantia+ Sedentaria).

## Methods

### Specimens

Specimens of *Magelona mirabilis* (Johnston, 1865) (Fig. 1a) were collected intertidally in the sandy flat of Pouldohan (Tregunc, Finistere), close to the city of Concarneau (Brittany, France) in March 2016 or in Morgat (Brittany, France) during June/July 2015. *Magelona alleni* Wilson, 1958 was collected in the bassin of Arcachon (France) in 1990.

Some adults from the animals collected in 2015 were directly fertilized in Roscoff (Brittany/ France), others were transferred to Bergen (Norway) and reared in a tempered sea-water cycle. Adults were fed with a mixture of yeast and grinded fish food. After artificial fertilization in filtered sea water (FSW) the developmental stages were reared at 18 °C in glass bowls containing 0.5 l FSW. The culture was not aerated, set under strict diurnal rhythm (14:10 – light: dark) and fed with a mix of unicellular algae (*Isochrysis*). Water was changed regularly.

### Parafin- histology

We used the methodology previously described by [9]. Four specimen of *Magelona mirabilis* were stained with Azan. Two were sectioned in cross section, two in sagittal sections. *Magelona alleni* was stained with Masson-Goldner Trichrome. Additionally one specimen of *Magelona mirabilis* was stained with Palmgrens silver staining.

### Immunohistochemistry

Anatomical details of developmental stages of *Magelona mirabils* (Johnston, 1865) were investigated in whole animal preparations using standard immunohistochemical staining protocols and a range of well-established antisera as neural markers. Every staining was carried out using at least 5 (for adults) or 15–25 specimens (for larvae) of each stage. Although the specificities of the employed antibodies have all been established in numerous invertebrates, we cannot fully exclude that a given antiserum may bind to a related antigen in the investigated specimens. We hence refer to observed labelled profiles as exhibiting (antigen-) like immunoreactivity (−LIR). Negative controls were obtained by omitting the primary antibody in order to check for antibody specificity and yielded no fluorescence signal.

Adult animals for immunohistochemical investigations were treated as described in [9].

Animals were incubated with antibodies against FMRF-amide (ImmunoStar, Hudson, WI, USA), acetylated α-Tubulin (Sigma-Aldrich, Saint Louis, MO, USA) and for the DNA staining Sytox (Invitrogen, Carlsbad, CA, USA). Preparations were scanned with a Leica TCS SPE CLSM. Image stacks were further processed using Fiji (1.52 h) [57].

For larval investigations we followed the protocol described in [18]. In detail, specimens were rehydrated from MeOH into PTW (PBS with 0.1% Tween 20), rinsed 2 × 5 min in PTW at RT (room temperature) and incubated in 10 μg proteinase K/ml PTW for 2–3.5 min. After rinses in glycine (2 mg glycine/ml PTW), and subsequent washes in PTW, the larvae were re-fixed using 4% PFA in PTW for 20 min at RT. Afterwards, all stages were rinsed twice in PTW, two times in THT (0.1 M TrisCl, 0.1% Tween) and incubated for 1–2 h in 5% sheep serum in THT. Subsequently, the samples were incubated in primary antibodies (polyclonal rabbit anti-serotonin from INCSTAR, Stillwater, USA, dilution 1:500; monoclonal mouse anti-acetylated α-tubulin from clone 6-11B-1, Sigma-Aldrich, St. Louis, USA, dilution 1:500; polyclonal rabbit anti-FMRFamide from ImmunoStar Inc., Hudson, USA, dilution 1:1000) for 24–72 h in THT containing 5% sheep serum at 4 °C. After incubation, specimens were then rinsed in 1 M NaCl in THT, 5 × 30 min in THT and incubated with secondary fluorochrome conjugated antibodies (goat anti-rabbit Alexa Fluor 488, Invitrogen, USA, dilution 1:500; goat anti-mouse Alexa Fluor 633, ANASPEC, Fremont, USA, dilution 1:500) in THT containing 5% sheep serum for 24 h. After successful incubation at 4 °C, the specimens were washed several times in THT, counterstained with DAPI for 10–15 min (5 mg/ml stock solution, working solution: 2 μl in 1 ml THT – final concentration 10 μg/ml) and washed 2 × 5 min in THT. Finally, the different larval stages were mounted between cover slips using 90% glycerol/ 10% 10x PBS containing DABCO and analysed with a Leica TCS SP5 (Leica Microsystems, Wetzlar, Germany). Confocal image stacks were processed with Leica AS AF v2.3.5 (Leica Microsystems), ImageJ and Imaris 9.3 (Bitplane AG, Zurich, Switzerland).

### μCT

Specimen were treated as described in [9]. Animals were scanned with a Skyscan 1272 (Burker, Germany) at 2 μm resolution. Image stacks were further processed using Fiji (1.52 h) [57]. 3D reconstructions and volume renderings were performed with Amira (5.0).

### Semi thin sectioning and TEM

We used the methodology previously described in [9]. Ultra- thin sections were analyzed in a ZEISS EM10CR transmission electron microscope. Semi- thin sections were analyzed using a light microscope (BX-51, Olympus).

### SEM

Specimen were treated as described in [9]. Animals were studied in a Philips XL30 ESEM.

### Data analyses and 3D-reconstruction

Living specimens were photographed with a Canon 600D Camera mounted on a Zeiss- Stemi 2000. Parafin- and semithin sections were analyzed with an Olympus microscope (BX-51). Sections were photographed with an Olympus camera (Olympus cc12) using the dot slide system (2.2 Olympus, Hamburg) and aligned using imod [58] and imod align (http://www.q-terra.de/biowelt/3drekon/guides/imod_first_aid.pdf). 3D reconstructions were performed with Fiji (1.45b) [57]/trakem [59] and Amira (5.0). Adobe (San Jose, CA, USA) Photoshop (CC) and Illustrator (CS6/CC) were used to prepare all figures.

### Data repository and voucher material

For data transparency, all aligned Azan-stained serial sections are freely available in MorphDbase: www.morphdbase.de [60–62]. The voucher material of species studied is deposited at the Institute of Evolutionary Biology and Zooecology of the University of Bonn.

*Magelona mirabilis*: http://www.morphdbase.de/?P_Beckers_20170310-M-92.1

*Magelona mirabilis* 2: www.morphdbase.de/?P_Beckers_20170627-M-99.1

*Magelona mirabilis* sagittal: www.morphdbase.de/?P_Beckers_20190318-M-105.1

*Magelona mirabilis* μCT part1: http://www.morphdbase.de/?P_Beckers_20190318-M-107.1

*Magelona mirabilis* μCT part2: www.morphdbase.de/?P_Beckers_20190318-M-106.1

### Orrhage’s sections

Some of the original sections that Lars Orrhage used for his reconstructions of the annelid nervous system are deposited at the Stockholm Naturhistoriska riksmuseet curated by Lena Gustavsson. We analyzed and photographed Orrhage’s sections of *Magelona papillicornis* [15] to evaluate our results and conclusions. Sections thickness is 4 μm and sections were stained with hematoxylin and eosin.

## Additional files

Additional file 1: *Magelona alleni*, histological cross sections (5 μm), Masson-Goldner Trichrome, frontal (A) to caudal (C). A: The brain (*br*) surrounds the frontally located coelomic cavities (*co*). Intermediate filaments (*if*) run through the neuropil. B: More posterior the brain consist of a dorsal part (*dpbr*) which gives rise to dorsolateral parts of the brain (*dlpbr*) which connect the dorsal brain to the ventral part of the brain. *mo*: mouth opening. C: More posterior the *cns* is composed of paired lateral medullary cord (*lmc*) which run caudally rectangular to the brain inside the epidermis (*ep*). Giant fibres (*gf*) initially are small. A cluster of enlarged neurons (*S3*) is present in the dorso- lateral part. *bl*: basal lamina; *if*: intermediate filaments. (PDF 10348 kb)

Additional file 2: comparison of Orrhage’s sections of *Magelona papillicornis* and *Magelona mirabilis* (this study). A, D, F: *Magelona mirabilis*, Azan, 5 μm. B, C, E: *Magelona papillicornis,* hematoxylin and eosin, 4 μm. (PDF 5597 kb)

Additional file 3: 3D- PDF of the 3D- reconstruction of the central nervous system of *Magelona mirabilis*. *Bright blue*: neuronal somata type 1; *cyan*: neuronal somata type 2; *dark blue*: neuronal somata type 3; *green*: lateral organ; *grey*: neuropil; *purple*: neurons of the lateral organ; *red*: palp nerves. (PDF 15703 kb)

## Data Availability

The voucher material of *Magelona mirabilis* and *Magelona alleni* is deposited at the Institute of Evolutionary Biology and Zooecology of the University of Bonn. All aligned serial sections, as well as μCt-scans are freely available in MorphDbase: www.morphdbase.de. *Magelona mirabilis*: www.morphdbase.de/?P_Beckers_20170310-M-92.1 *Magelona mirabilis* 2: www.morphdbase.de/?P_Beckers_20170627-M-99.1 *Magelona mirabilis* sagital: www.morphdbase.de/?P_Beckers_20190318-M-105.1 *Magelona mirabilis* μCT part1: www.morphdbase.de/?P_Beckers_20190318-M-107.1 *Magelona mirabilis* μCT part2: www.morphdbase.de/?P_Beckers_20190318-M-106.1

## Abbreviations

*BG1-BG3*: erstes, zweites, drittes Bauchganglion
*cc*: circumesophageal connectives
*cns*: central nervous system
*DG*: Dorsalganglion auf hinterer Schlundkommissur
*dKhS*: dorsale Kommissur des hinteren Schlundkonnektivs
*dKvS*: dorsale Kommissur des vorderen Schlundkonnektivs
*dpf*: days post fertilization
*hg*: hinteres Ganglion
*hs*: hinteres Schlundkonnektiv
*PN°*: oberer Palpennerv
PN^*u*^: unterer Palpennerv
*S1-S3*: different neuronal somata, differentiated by size, S1 small- S3 large
*vKhS*: ventrale Kommissur des hinteren Schlundkonnektivs
*vKvS*: ventrale Kommissur des vorderen Schlundkonnektivs
vS: vorderes Schlundkonnektiv
