# Supplementary figures and images for: The anatomy and development of the nervous system in Magelonidae (Annelida) – insights into the evolution of the annelid brain
Source: BMC Evol Biol. 2019 Aug 28;19:173. doi: 10.1186/s12862-019-1498-9 (PMC6714456; doi:10.1186/s12862-019-1498-9)

**A**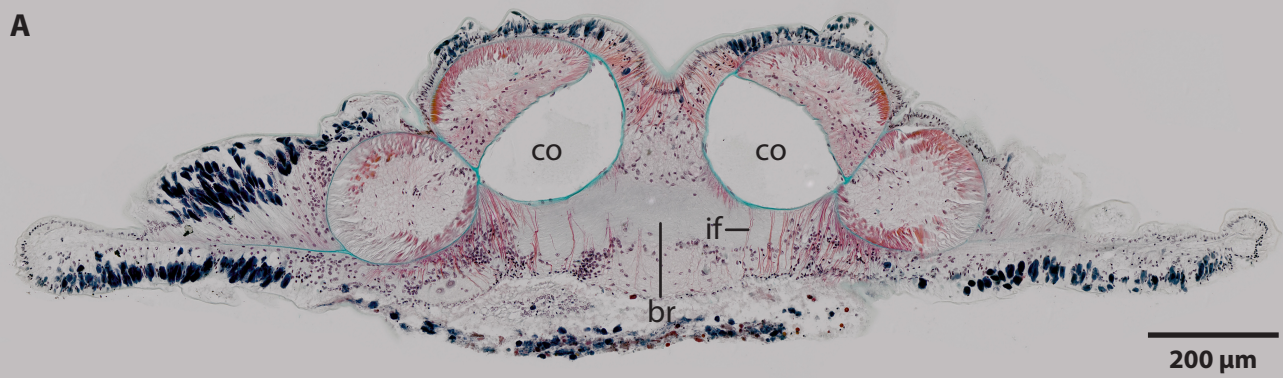**B**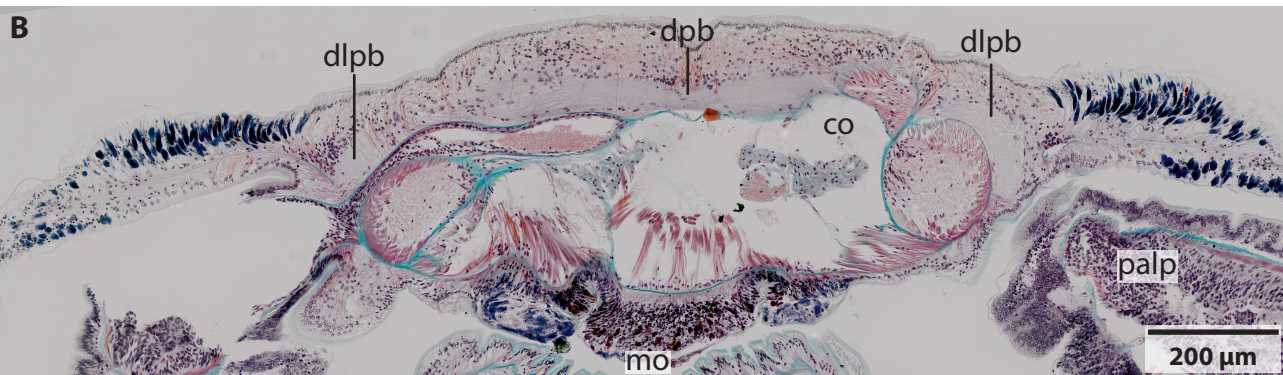**C**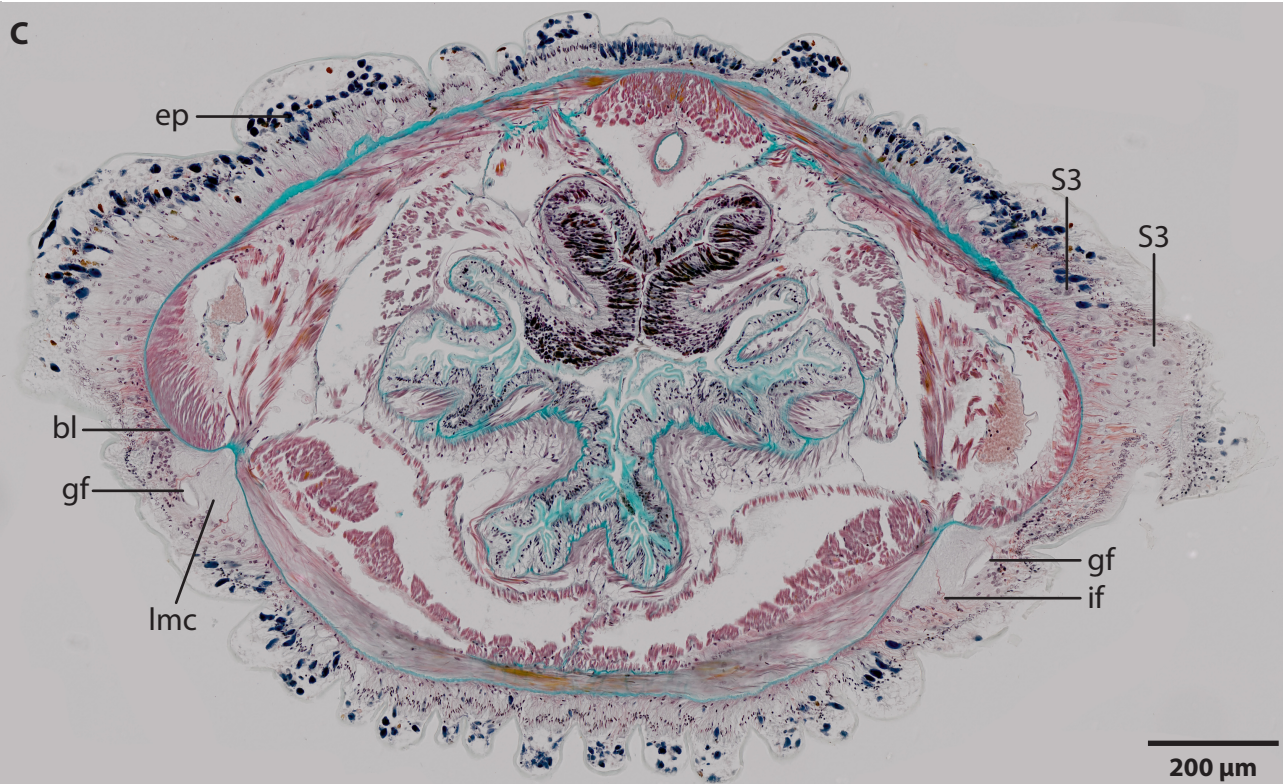

Supplement: Supplementary file 1 — Magelona alleni, histological cross sections (5 μm), Masson-Goldner Trichrome, frontal (A) to caudal (C). A: The brain (br) surrounds the frontally located coelomic cavities (co). Intermediate filaments (if) run through the neuropil. B: More posterior the brain consist of a dorsal part (dpbr) which gives rise to dorsolateral parts of the brain (dlpbr) which connect the dorsal brain to the ventral part of the brain. mo: mouth opening. C: More posterior the cns is composed of paired lateral medullary cord (lmc) which run caudally rectangular to the brain inside the epidermis (ep). Giant fibres (gf) initially are small. A cluster of enlarged neurons (S3) is present in the dorso- lateral part. bl: basal lamina; if: intermediate filaments. (PDF 10348 kb) [file 12862_2019_1498_MOESM1_ESM.pdf]

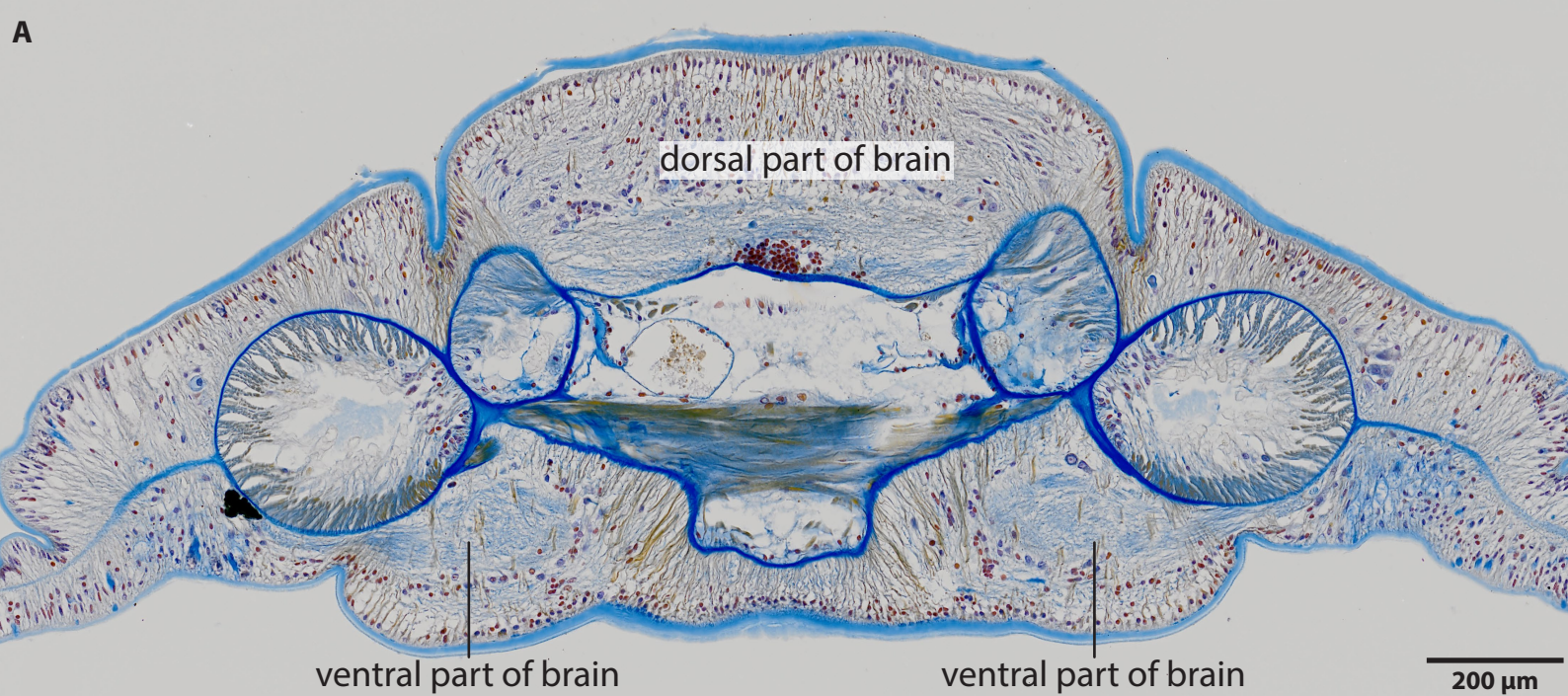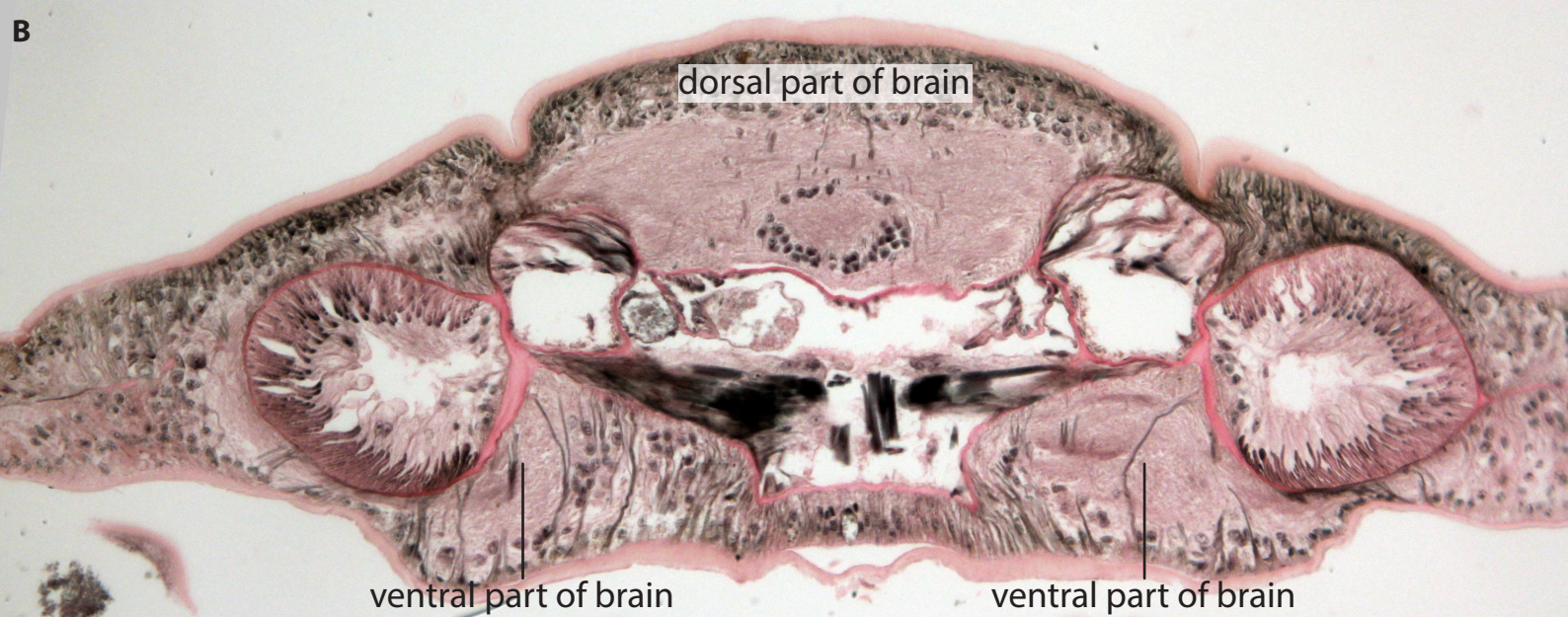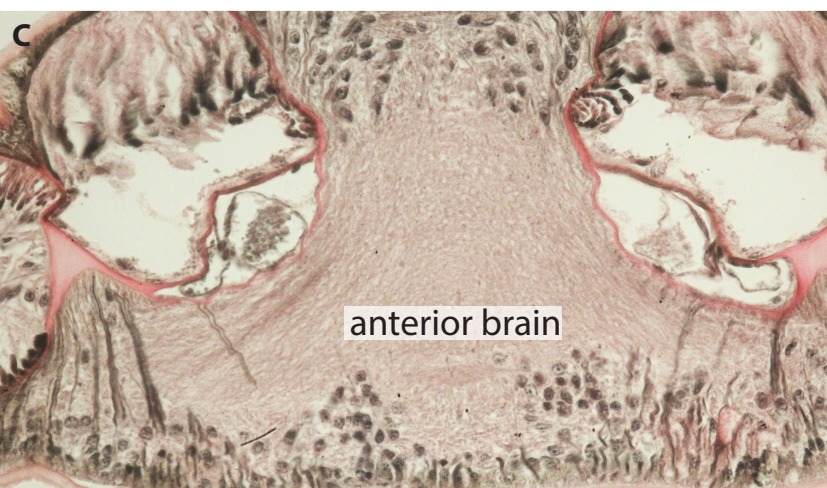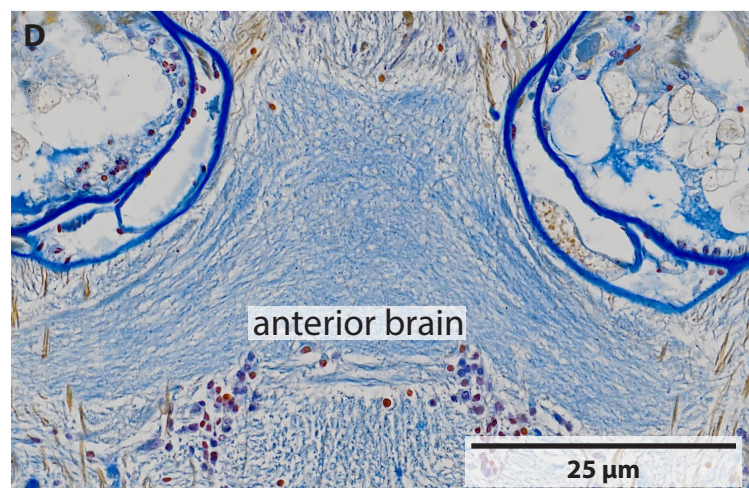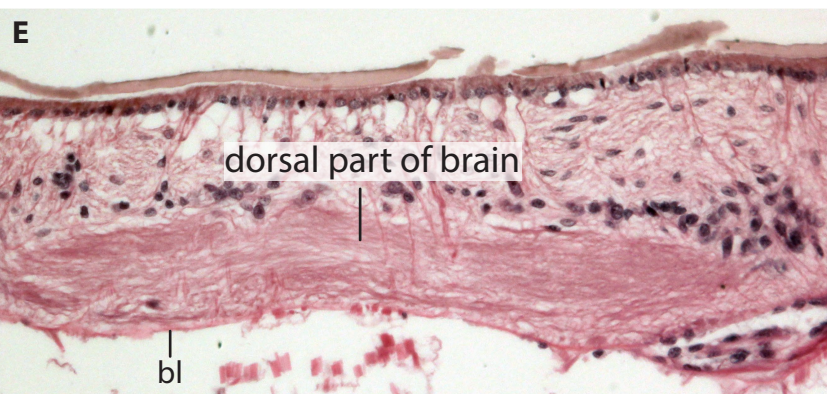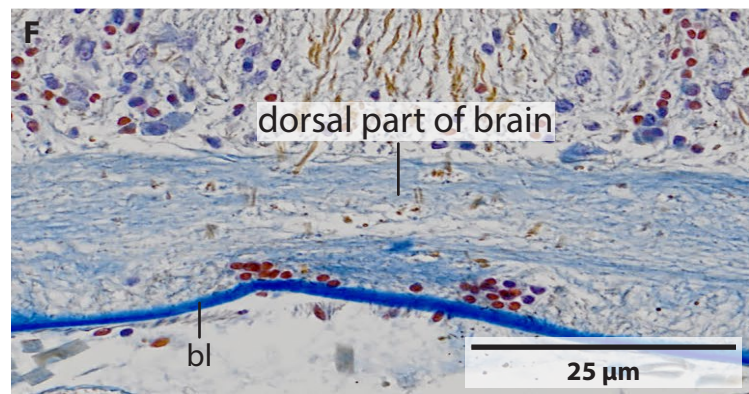

Supplement: Supplementary file 2 — comparison of Orrhage’s sections of Magelona papillicornis and Magelona mirabilis (this study). A, D, F: Magelona mirabilis, Azan, 5 μm. B, C, E: Magelona papillicornis, hematoxylin and eosin, 4 μm. (PDF 5597 kb) [file 12862_2019_1498_MOESM2_ESM.pdf]

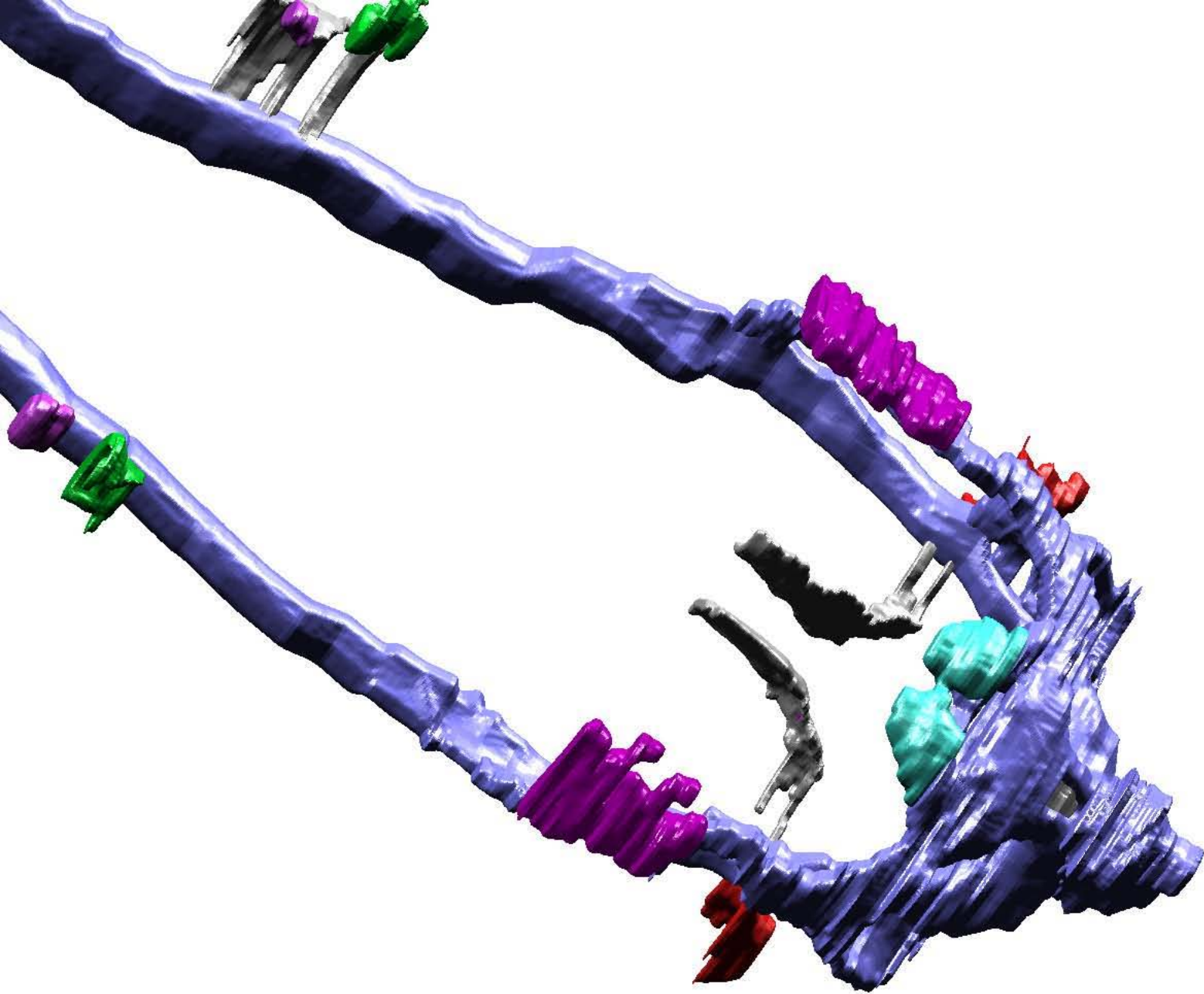

Supplement: Supplementary file 3 — 3D- PDF of the 3D- reconstruction of the central nervous system of Magelona mirabilis. Bright blue: neuronal somata type 1; cyan: neuronal somata type 2; dark blue: neuronal somata type 3; green: lateral organ; grey: neuropil; purple: neurons of the lateral organ; red: palp nerves. (PDF 15703 kb) [file 12862_2019_1498_MOESM3_ESM.pdf]
